# The crisis in American science

**DOI:** 10.1177/00732753251343655

**Published:** 2025-05-29

**Authors:** Kathryn M. Olesko, Anthony Eames, Cyrus C. M. Mody, Ilana Löwy, Thomas Zeller, Mark Walker, Mitchell G. Ash, Donna Haraway

**Affiliations:** Georgetown University, USA; George Washington University and Foreign Policy Research Institute, USA; Maastricht University, Netherlands; Centre de recherche médecine, science, santé et société, France; University of Maryland, College Park, USA; Union College, USA; University of Vienna, Austria; University of California, Santa Cruz, USA

**Keywords:** Science in America, science and ideology, federal science, response and resistance to regime, Department of Government Efficiency, Executive Orders, Third Reich, brain drain, tacit knowledge, Silicon Valley oligarch

## Abstract

The second regime of Donald J. Trump has decimated federal science and federally supported science to a greater degree than his first regime, unleashing an unprecedented crisis. The authors in this roundtable take a close look at the tortured landscape of science in America and, using historical scholarship and the tools of history, analyze and assess its plight. Topics include nuclear facilities, the role of Silicon Valley oligarchs, Executive Order 14168 on biological sex, climate change and the environmental sciences, science and ideology, responses to regime change, and resistance to the regime, especially by universities.

## Introduction

**Kathryn M. Olesko**

Georgetown University, USA

Times of crisis galvanize historians, who see the present through the lenses of the past. Now is one of those times when, tragically, the scientific community in the United States is again under attack from the federal government, the most important patron of science in America. This special roundtable commentary on the crisis in American science was assembled at the request of the Editor of *History of Science*, Lissa Roberts. With its long tradition of historical scholarship coupled with historiographical reflection, *History of Science* is a particularly appropriate venue for analyzing this crisis. The challenges facing historians who address this crisis are considerable. Donald J. Trump’s treatment of science in many respects repeats his actions during his first regime, 2017–21, but not entirely. What appear to be other historical precedents in American history prove to be in one sense or another incomparable. International comparisons with totalitarian regimes seem more relevant than past eras of American history. And the complexion of circumstances that encase this current crisis are without historical parallel. In their analyses of the current state of science in America, the authors in this roundtable leverage history of science and, more broadly, science studies to examine more deeply what has happened and to assess the likely unfortunate consequences of the cascading changes to the scientific landscape. They zoom in on the dangers caused by senseless budget cuts to U.S. nuclear energy and waste programs, the administration’s misuse of science to eviscerate recognition of and equality for nonbinary peoples, and its destructive targeting of any research related to climate change and the environment. They remind us of the facets of Silicon Valley’s history that have been obscured by attention to the reactionary culture championed by Peter Thiel and his ilk; draw our attention to the relative stability of science under past authoritarian regimes compared to the chaos that reigns in America now; ask us to focus on the power politics at work in the struggle between science and the federal government; and resurrect the long history of collective resistance to the federal government to deter us from thinking of obedience as a first response. Together they call on professional historians to take up our moral obligation and raise our voices now before it is too late. The hope is that this effort stimulates other conversations on the current crisis and on the all-important relationship between the past, present, and future of science in America – and elsewhere.

## The topography of the crisis

The United States is in the midst of an unprecedented, hostile, intentionally unlawful takeover of its government by members of a regime who are closely following the far-right-wing playbook Project 2025. This now infamous manifesto purports to provide a roadmap for achieving conservative social, political, economic, and cultural goals based on adaptations of the draconian and illiberal strategies of other authoritarian regimes, notably Viktor Orbán’s in Hungary.^1^ A special target of the project is bureaucratic expertise, which is regarded with contempt. Russell T. Vought, one of the project’s architects who is now director of the U.S. Office of Management and Budget, is reported as wanting to “demonize civil servants, which include scientists and subject matter experts.” In his own words: “We want the bureaucrats to be traumatically affected. When they wake up in the morning, we want them to not want to go to work because they are increasingly viewed as the villains. We want their funding to be shut down so that the EPA [Environmental Protection Agency] can’t do all of the rules against our energy industry because they have no bandwidth financially to do so.” He ends: “We want to put them in trauma.”^2^

And they did. In the 100 days since the presidential inauguration on January 20, 2025, the regime of Donald J. Trump, aided by bureaucratic loyalists and especially the congressionally unvetted Department of Government Efficiency (DOGE), has fired federal scientists, diminished or closed several scientific divisions of federal agencies, reduced or eliminated budgets for scientific research in the federal government, and suspended federally funded grants for science and scholarship that were competitively awarded to universities and other institutions. Science has suffered before as a result of the changing winds of politics in Washington, including under Trump’s first regime.^3^ Now, however, even more damage has been inflicted on federal science and federally supported science in the past three months than in any other period in their history.

The targeting of science in the federal government began on Inauguration Day. A flurry of Executive Orders (EOs) terminated diversity, equity, and inclusion programs, which had formerly assured equitable treatment for hiring and grants (EO 14151); ended certain environmental protections (EOs 14153,14154, 14156, 14162); withdrew the United States from the World Health Organization, thereby cutting off access to data and ending American contributions to that data, which meant, inter alia, that the United States would not be receiving or sharing information on pandemics and epidemics, putting Americans at risk (EO 14155); and established the DOGE, whose leader and minions have proven to be woefully ignorant of how the federal government actually works (EO 14158).^4^ The most scientifically stupefying was EO 14168: Defending Women from Gender Ideology Extremism and Restoring Biological Truth to the Federal Government.^5^ Trump has been known to contest scientific truth before, as when he promoted the antimalarial drug hydroxychloroquine as a prophylactic against and treatment for Covid, which has now been linked to an estimated 17,000 deaths.^6^

These eight EOs created the skeletal framework for what followed in the administration’s attacks on science over the next 100 days. A day after the inauguration, EO 14173 went into effect. It barred supporting any initiative that was offensive to the president’s policy prerogatives, such as climate change and anything related to diversity, equity, and inclusion, including studies of how to recruit more women and minorities into the sciences.^7^ Three days after the inauguration EO 14177 established the President’s Council of Advisors on Science and Technology, the agents charged with correcting what the president viewed as the wayward course of science and technology:

At the heart of scientific progress lies the pursuit of truth. But this foundational principle, which has driven every major breakthrough in our history, is increasingly under threat. Today, across science, medicine, and technology, ideological dogmas have surfaced that elevate group identity above individual achievement, enforce conformity at the expense of innovative ideas, and inject politics into the heart of the scientific method. These agendas have not only distorted truth but have eroded public trust, undermined the integrity of research, stifled innovation, and weakened America’s competitive edge.^8^

In the days that followed, the public learned how these EOs would be carried out to decimate and restructure federal scientific institutions.

While not federal laws, EOs do have the force of law in agencies that fall under the aegis of the executive branch, including the Department of Defense (DOD), where there is a significant scientific and engineering presence. So, it is not at all surprising that under the umbrella excuse of reducing the federal budget and the budget deficit, Cabinet secretaries (especially Robert F. Kennedy, Jr. of Health and Human Services [HHS] and Pete Hegseth of DOD) and other federally appointed directors (primarily Elon Musk of DOGE) have seized the opportunity to prune science in the federal government and federally supported science. Few have noticed, though, that strictly speaking, EOs cannot be used to eliminate, appropriate, or reassign funds that Congress, the legislative branch, had already approved for specific purposes. Nevertheless, the mostly young DOGE staff drawn from Musk’s inner circle descended on federal agencies, sidelined agency directors, infiltrated computer systems, and began to ruthlessly identify budget cuts and dismantle the infrastructure of federal science.

Every single center of scientific research in the federal government has been subject to funding and staff reductions. These cuts appear to be arbitrary and based on a profound ignorance of the most sensitive scientific and technological operations in the federal government. Some of the most salient and egregious cuts present real dangers, present and future. Take the National Nuclear Security Administration (NNSA), overseen by the Department of Energy. DOGE summarily fired 300 workers at NNSA in mid-February 2025, not realizing the agency was responsible for safeguarding and maintaining America’s stockpile of 3,748 nuclear bombs, including such critical tasks as reassembling warheads, which is necessary for the modernization of America’s nuclear arsenal. The agency already had problems recruiting and retaining a specialized workforce that required intensive on-the-job training and top-secret Q clearances; the cuts added to its woes. Days later when the mistake was realized, the dismissals were reversed, but the damage was already done. A month later, the agency was still understaffed because the federal government had, in addition, offered early retirements, including to seasoned nuclear arms control experts.^9^ Occurring at a time when the United States is trying to resume nuclear talks with Iran, the losses impacted not only national security, but international security as well.

Some of the cuts are targeted retaliations. The National Institutes of Health (NIH), regarded as the premier health research agency globally, has repeatedly been in the crosshairs of both Trump administrations. In what can only be regarded as an act of retribution, the leadership of NIH’s National Institute of Allergy and Infectious Diseases, directed from 1984 to 2022 by Anthony Fauci – whom the president despises due to Fauci’s justified attempts to control the scientific and medical narratives of the Covid pandemic – was eviscerated. The revenge did not end there. The administration fired Christine Grady, a prominent NIH bioethicist and wife of Anthony Fauci, and offered her instead a position in Alaska’s Indian Health Services. The same offer was extended to scientists at the Food and Drug Administration (FDA) and the Centers for Disease Control and Prevention (CDC), both sites of medical viewpoints at odds with the administration’s contrary ones.^10^ Just as the administration is sending supposed aliens to a gulag in El Salvador, it is displacing unwanted intellectuals to the chilly frontier, mirroring the practices of the Soviet Union and Russia.

The NIH, FDA, and CDC fall under HHS, which is expected to lose more than 10,000 staff members over the next few months, with dire consequences for the American public.^11^ Already at the FDA, staff layoffs have led to a decline in clinical trials and delays in drug development, and in the CDC, entire investigative units covering public health issues have been eliminated, including the Office of Smoking and Health. Infectious diseases are likely to be the primary focus of the CDC, but one with a dangerous position on vaccines due to the antivaccine stance of HHS’ secretary, Robert F. Kennedy, Jr.^12^

As a former member and director of the Program in Science, Technology, and International Affairs at Georgetown University – a position where I became immersed in Cold War and contemporary American science and learned how to apply history to policy – I am dumbfounded by the administration’s erasure of the social sciences that have informed domestic and international policy decisions. DOD’s Minerva Initiative, which was established as a result of the policy failures of 9/11 and had supported creative academic work in the social sciences pertinent to military planning and intelligence, was terminated.^13^ Social science work supported by the Pentagon was crucial for assessing emerging threats to national security. The Woodrow Wilson International Center for Scholars, which was established by Congress and has a Science and Technology Innovation Program, offered fellowships to intellectuals of all stripes for the purpose of advancing the role of the social sciences in policy making. The Center lost its federal funding and much of its staff, and has been reduced to maintaining only its minimum statutory functions, offering only a few annual fellowships henceforth, but not enough to maintain any sense of programming and dissemination of its scholarship.^14^ This dismissal of the social sciences not only hurts policy making in the federal government. It also hurts academia: new insights fostered in these institutes have fed back into academic curricular innovation.

These are just the tip of the iceberg of massive and tragic eviscerations of the scientific and technological infrastructure of the United States that have occurred over the past three months. Other agencies – including the National Aeronautics and Space Administration, the National Geospatial Intelligence Agency, the National Institute of Standards and Technology, the National Oceanic and Atmospheric Administration (which houses the National Weather Service and had supported climate-change studies), the National Science Foundation, the U.S. Geological Survey, and more – have also been severely cut in size and budget.^15^

The damage that has already been done is compounded by the lack of attention paid to their consequences for scientific training, and so for the future of the American scientific enterprise. Many of the grants awarded by federal agencies include both graduate and postdoctoral fellowships. When these grants are reduced, the pipeline of future scientists is narrowed considerably. Of deep consequence for generational reproduction in the sciences is the inexplicable reduction by half of the prestigious National Science Foundation (NSF) graduate fellowships. Over time the number of NSF fellowships has varied, but generally the number has grown, most recently to 2,555 in 2023, a number that was reduced by twenty percent in 2024 to 2,000. The 2025 reduction to 1,000 fellowships brings the number to its lowest in fifteen years; the number during the first Trump regime was higher. The reduction led immediately to a curtailment in graduate admissions in the sciences for the academic year 2024/25 because it occurred before admission offers were mailed in the spring 2025 semester.^16^

What does history have to say about these developments?

First, the obvious point of historical comparison is Trump’s science policy during his first regime, 2017–21, when his primary targets were the environmental sciences, climate change, and later during the pandemic, health measures related to Covid. Science became politicized, conformity to the administration’s ideas about science was expected (especially regarding Covid), and the administration openly interfered with the dissemination of scientific results in order to prevent the circulation of information contrary to the administration’s beliefs (climate change does not exist) and policies (promoting unproven drugs for the treatment of Covid).^17^ Paradoxically, Trump rarely takes credit for Operation Warp Speed, which resulted in effective Covid vaccines in record time through a public–private partnership.^18^ Are his actions thus far in 2025 comparable to those in 2017–21? Yes and no.

In 2017–21 and now, the executive branch decimated several federal science agencies; canceled federal research grants; destroyed data sets, especially on climate change; defunded climate science and eliminated environmental protections; and imposed censorship on scientists who held views contrary to the administration’s. But there are considerable differences that thus far separate the two regimes. Trump did not have a master plan for science policy before; Project 2025 guides him now. He was criticized earlier for axing federal science advisory committees and not filling science advisory positions fast enough; now he has them, questionable though their credentials may be. Few technology oligarchs were by his side in his first regime, while now they slavishly support him and benefit substantially from federal contracts and other favors. Now he has extended his reach deeper into the scientific and medical enterprise. He has used an EO to impose a scientifically groundless definition of sex and, by implication, gender; cultivated doubts about vaccines via bureaucratic appointments; and deepened a contempt for expertise, including in policy matters, in order to eliminate the opposition by fiat.^19^ Perhaps the biggest difference between then and now is the presence of his personal demolition agency, DOGE.

Disturbing compounding factors further threaten the scientific enterprise now. One is the administration’s attempt to control academic medical journals so that “competing viewpoints” are represented to eliminate what is perceived as “political bias” in articles selected for publication, thereby subverting the standards-driven academic refereeing process and casting a chill over knowledge production in academia. This attempt at censorship follows hard on the heels of other administration officials demonizing the gatekeeping function of journal boards and editors, calling it a form of “groupthink,” thus challenging scientific consensus.^20^ Another more insidious development is the administration’s concurrent assault on institutions of higher learning, which the administration seeks to control by abrogating their First Amendment rights and the professional right to academic freedom, and by threatening them with the repeal of federal grants and their tax-exempt status as nonprofits. In line with Project 2025, the intent is to remake higher education, the principal home of scholarship in America.^21^ There is no analogous point of comparison in American history for the simultaneity of these executive actions that have imperiled science, even though there are several parallels in Trump’s first regime.

The overall damage inflicted by the current administration on the scientific enterprise has been deep and wide-ranging. Enacted rapidly without forethought, study, or reasoned assessment of needs and impact, the administration’s actions targeting science seem to be guided by arbitrary budget, facility, and workforce reductions, ideological preferences, and at times, a compulsion to retaliate against perceived enemies, both personal and intellectual. Authors Anthony Eames, Donna Haraway, Ilana Löwy, and Cyrus C. M. Mody analyze several of these actions in this roundtable commentary, using the toolboxes of history and science studies to draw out their implications and consequences.

Second, other earlier historical precedents for some of the executive branch’s actions offer only weak comparisons. The current Trump administration has tried initially to impose a cap of 15% on indirect costs on NIH and other federal grants. This attempt was not entirely out of line with past practices, as indirect costs were capped between 1947 and 1965 at between 8% and 25%. When the cap was removed in 1966, indirect costs were negotiated between federal agencies and university administrations, but periodically Congress or the president would try to lower them for budgetary reasons because they had increased. An injunction was placed on the Trump administration’s proposal, however, and to date, the administration has not challenged it for reasons unknown, so the issue of indirect costs is moot.^22^ Concerning federal reductions in force, Elon Musk and his DOGE brethren and sisters have argued that President Bill Clinton’s reductions in force during the 1990s is a model for what DOGE is doing. That’s simply not true. Clinton used the rules in place for reduction in force and, with Vice President Al Gore at the helm, conducted a National Performance Review that took six months to complete before any reduction proposals were made. Moreover, the entire effort was sensitive to the need for congressional approval where it was necessary. In the end, Clinton’s buyouts had strong bipartisan congressional support. With them and other budgetary changes, Clinton was moreover able to balance the budget thanks to his National Partnership for Reinventing Government.^23^ Finally, the anticommunist McCarthy era from the late 1940s through the 1950s offers a parallel moment when the federal government attacked the left and persecuted scientists and other intellectuals after imposing ideological litmus tests. J. Robert Oppenheimer lost his security clearance and scientists, like mathematician Chandler Davis, left the United States for Canada. One major difference, though, between then and now is that between the late 1940s and 1959, federal obligations for research and development grew, and continued to do so until 1967.^24^

Third, American history fails to offer a point of comparison for the occupation of the Oval Office by an individual who, as a convicted felon, blatantly and openly cultivates, with the support of political appointees approved by Congress and without significant congressional objection, an authoritarian regime and friendships with other dictators, especially Russia, the long-time enemy of the United States. Instead, comparisons with other similar regimes, including the Third Reich and the Soviet Union, seem to be more fitting. During the first Trump regime, historians began to question how close the United States was to Germany in 1933 when Adolf Hitler came to power. “Closer than we think” was the answer.^25^ Similar questions were raised during the second Trump run for office, before the November 2024 American election, even by the very conservative *Wall Street Journal*.^26^ Can similar comparisons be made now about how these regimes treated science? In this roundtable, Mitchell G. Ash, Mark Walker, and Thomas Zeller address this question, arguing that comparisons to science in Nazi Germany or the Soviet Union, while appropriate and revealing, do not offer parallels as much as accentuate the exceptionality of what is happening to science in the United States.

The administration’s actions have stymied scientific discovery and innovation and demoralized the scientific community. With funding for graduate fellowships reduced or nonexistent, students are reconsidering their plans to commit to a career in science.^27^ These sentiments cut so deep that *Science*, the weekly journal of the American Association for the Advancement of Science, published an editorial that admonished scientists: “don’t quit the long game.”^28^ Nonetheless, scientists have begun to think of moving elsewhere. Advertisements have appeared in other countries that view American scientists as sources of enrichment for their own scientific communities and agendas.^29^ Within a month of the presidential inauguration, the Max Planck Society (Max-Planck-Gesellschaft), Germany’s premier set of research institutes, identified American scientists as a “new talent pool.” Germany has targeted for recruitment specifically those areas that the current administration has gutted: climate change and environmental science, Earth system science, gender studies, and infectious disease research. Patrick Cramer, the president of the society, criticized the administration’s defunding of research areas as “a clear violation of academic freedom, for the choice of research topics is up to the researchers, not the White House.”^30^ The decline of support for science portends disaster not only for its future and the scientific profession in America, but also for global science, according to Otmar Weistler, president of another German research powerhouse, the Helmholtz Association (Helmholtz-Gemeinschaft Deutscher Forschungszentren), Germany’s most well-funded research organization. “The greater the interference in science,” Weistler noted, “the more intense the global consequences will be, including for the [United States] itself.”^31^ This brain drain just adds to the diaspora of scientists from the federal government that began during Trump’s first regime, signaling a further decline in America’s global position in science.^32^

Stand up for science!

April 19, 2025

The 250^th^ Anniversary of the American Revolution

## Courting a normal accident: The DOGE risk to nuclear energy and waste policy

**Anthony Eames**

George Washington University and the Foreign Policy Research Institute

The bone-deep cuts levied by the Department of Government Efficiency (DOGE) on the nuclear enterprise demonstrate a flawed understanding of how that enterprise works and of the lessons of its past history. While Donald Trump and Elon Musk have aggressively targeted supposed waste in the federal workforce and budget, they ignore the safe handling of a far more dangerous type of waste, the radioactive variety. Gutting the expertise and institutions responsible for the safe handling of nuclear materials risks the viability of a much-needed nuclear energy revival, which is gaining bipartisan support amid the rise of artificial intelligence (AI) and increasing electricity demand.

Largely neglected by the Atomic Energy Commission for the first three decades of the nuclear age, problems of radioactive waste drew public attention with the partial meltdown at Three Mile Island in 1979. For the Reagan administration, the lingering public opposition to nuclear power as a result of that accident, combined with shocks of the 1970s to oil markets, made direct government support for the flagging commercial nuclear energy industry all the more important. In a move that ran counter to the Republican philosophy of limited government, the administration’s initiatives to invigorate the nuclear energy industry resulted in the 1982 Nuclear Waste Policy Act, shifting the responsibility of radioactive waste storage – both high-level waste and spent nuclear fuel – onto the federal government. Nine years later the Nunn-Lugar Act for Cooperative Threat Reduction (1991) led the U.S. government to assume partial responsibility for securing fissile material in the former Soviet republics and, eventually, around the world. This legislation dramatically expanded the U.S. government’s responsibility for managing radioactive material produced by private enterprise and foreign nations. They were championed both by conservatives in the Congress and by Republican presidents, who otherwise fully embraced the idea of limited government.

The nuclear energy industry is uniquely dependent on the federal government to clean up its waste byproducts at home, while abroad the United States is indispensable to nonproliferation efforts that limit the accessibility of fissile material to bad actors. These missions depend on the expertise and independence of the nuclear security enterprise that is spread across a largely unknown alphabet soup of agencies. Until the recent spate of panicked firings and rehirings, most Americans had likely never heard of the National Nuclear Security Administration (NNSA), the agency charged with stockpile stewardship and the refurbishment of nuclear weapons. Fewer still have probably heard of the Waste Isolation Pilot Plant, the only permanent geological repository for transuranic waste in the United States, or the Defense Threat Reduction Agency (DTRA), which has safely removed thousands of tons of highly enriched uranium, plutonium, and other material from locations as diverse as the former Soviet Union, Japan, Guatemala, Iraq, and the United Kingdom. Cuts to these agencies’ facilities, staff, and operational budgets will make them more likely to face a series of crises in nuclear waste and materials management that science studies scholars identified long ago.

Take, for example, the problem of tacit knowledge. Thirty years ago, Donald MacKenzie and Graham Spinardi identified the essential role of tacit knowledge in nuclear work. Technical intuition in the design of nuclear weapons is a requirement due to the difficulties in acquiring empirical data from nuclear testing to inform theoretical models; in the challenges in creating nondestructive tests to assess the bonds mating warheads to delivery vehicles; and in the search for material imperfections in the quality control processes for component parts. In other words, managing nuclear materials is both art and science.^33^ Younger technical personnel rely on the experience of their older colleagues.

That experience is now being shown the door. Dozens of “star performers,” who oversee the production of plutonium pits, who manage the enrichment of uranium, who safely transport nuclear materials across the United States, who monitor environmental contamination at the site of bomb assembly, or who safely handle nuclear waste and materials on a daily basis in the United States are gone. They cannot so easily be replaced. Training experts in the nuclear enterprise – especially the imparting of tacit knowledge – is a time-consuming endeavor. Officials have recently stated that junior employees at NNSA take at least a year to train.^34^ Training lags compound the problem of a long-running shortage in the nuclear workforce, which is – especially in the public sector – relatively older compared to other highly sensitive technical industries.^35^ While the latest cuts to the public nuclear workforce have undermined stability and work culture incentives, young talent sees opportunities in the private nuclear energy industry that is expected to triple its production capacity by 2050.^36^

The expansion of the nuclear energy industry is a rare issue with bipartisan support. Green groups view increased reliance on nuclear energy as necessary for achieving net-zero carbon emissions goals by 2050. AI advocates see small modular reactors (SMRs) as critical to powering data-center needs. In these prospects lies the possibility for a tacit knowledge crisis to converge with the problems presented by the reflexive modernity that characterizes a “risk society,” such as the unbinding of science and politics that leads to an erosion of trust in expertise and a systematic acceptance of higher levels of risk.^37^ For instance, should the SMR solution be embraced for the coming AI boom, the nuclear waste problem will become even more pronounced. The latest research on current designs for SMRs shows that they generate more nuclear waste per unit of energy than conventional plants. Moreover, they produce plutonium with a longer radiotoxic half-life than conventional plants.^38^ These concerns are increasingly subordinated to the enthusiasm for AI and corresponding energy needs. According to a recent survey of its members from the Nuclear Energy Institute, the major industry association representing the interests of commercial nuclear energy before the federal government, approximately 300 SMRs are expected to come online by 2050.^39^ Current practice is for SMRs, like their larger conventional brethren, to store nuclear waste on site. As of 2020, only eighty sites across the country stored nuclear waste; adding 300 more by 2050 without a corresponding growth in waste management workforce presents a serious problem.^40^

Demands for increased nuclear energy alongside cuts to the regulations and workforce that secure nuclear waste raise the question: Are industry and the U.S. government setting the conditions for what Charles Perrow calls a “normal accident”?^41^ Normal accidents occur in highly complex technological systems where components are tightly coupled with little slack, meaning that a single point of failure can cascade into disaster. They are by nature difficult to predict, but the most effective defense against accidents are well-trained and experienced operators who can instinctively navigate such complexities.

With the resounding bipartisan passage of the ADVANCE Act in 2024 during the Biden administration, Congress intended to stimulate a growth in the building, licensing, and operation of nuclear reactors not seen since the 1970s.^42^ The Trump administration has given an additional boost to industry interests by subordinating the independence of the Nuclear Regulatory Commission (NRC) to the Office of Management and Budget to ensure alignment with presidential priorities.^43^ Those priorities, recently expressed in the administration’s executive order establishing a National Energy Dominance Council, include “rapidly facilitating approvals for . . . Small Modular Reactors.”^44^ No SMR has ever been built and operated in the United States, yet, paradoxically, SMRs have been used to justify a rollback of safety regulations. Yet they could lead to problems. Nuclear reactors of identical design have been known to behave or, rather, “breathe” differently based on different environments and operators. Modularity does not equate to safety.^45^

Climate change further complicates this picture. SMRs that rely on passive safety systems may be uniquely vulnerable to extreme heat, floods, or other weather disruptions.^46^ The rollback of NRC safety inspections in 2019 – also enacted by Trump – and a shrinking, overstretched workforce only heighten this vulnerability. The result is a system with greater complexity, fewer buffers, and a diminished capacity to adapt – precisely the conditions that make a normal accident of the type discussed by Perrow more likely.^47^

Concerns about nuclear terrorism have been relatively absent in the SMR conversation. The failure to develop an alternative to the Yucca Mountain geological repository – alternatives opposed by the Obama, Trump, and Biden administrations due to significant site flaws that have revealed themselves since designation of Yucca Mountain as the sole commercial use repository in the 1987 Nuclear Waste Policy Amendments Act – means that dispersed nuclear waste storage will continue to be a reality for the foreseeable future. Earlier instances of government interventions to spur the growth of the nuclear energy industry, for example Richard Nixon’s “Project Independence” or George W. Bush’s “Nuclear Power 2010,” went hand-in-hand with concerted efforts to mitigate the increased potential for nuclear terrorism due to an ever-growing list of radiological targets and the recent memory of major terrorist attacks.^48^ This recognition does not appear to be the case in 2025 with DOGE reportedly setting its sights on the DTRA, an outfit that plays a critical role in the design and implementing of nuclear terror exercises.

In complex systems, people are the most adaptive and responsive components. A well-trained, adequately staffed nuclear workforce – across both public and private sectors – is our most effective defense against cascading failures. As the U.S. government accelerates the growth of nuclear energy while cutting the very institutions designed to manage its risk, it gambles with hazards that science studies scholars have repeatedly identified. Given nuclear energy’s unique susceptibility to NIMBY syndrome, keeping the industry accident free is the best way to support its growth. A renewed commitment to institutional memory, independent oversight, and tacit knowledge – not a blunt drive for “efficiency” – is necessary for the SMR renaissance to unleash progress rather than peril.

## Make Silicon Valley boring again!

**Cyrus C. M. Mody**

Maastricht University, Netherlands

Much was made during the 2024 presidential campaign of the “weird” beliefs held by JD Vance, Peter Thiel, David Sacks, Jeff Bezos, and Donald Trump’s other Silicon Valley allies. Musk, famously, believes we all live in a computer simulation.^49^ Bezos thinks he’ll achieve immortality.^50^ Thiel claims women’s suffrage initiated the West’s decline; and his college acquaintances insist that he supported apartheid in the 1980s.^51,52^ Thiel is also a fan of building libertarian sea colonies, prepping for the apocalypse, destroying news outlets he doesn’t like, and circulating young people’s blood through his veins to rejuvenate his cells.^53^ And by all accounts he’s one of the more grounded tech moguls!

Journalists and dissenting insiders have done much to reveal the Silicon Valley elite’s profoundly alternative reality. Yet historians of science and technology have done their part too, going back at least to Fred Turner’s 2006 book, *From Counterculture to Cyberculture: Stewart Brand, the Whole Earth Network, and the Rise of Digital Utopianism*.^54^ The years since have seen many fascinating studies of the “groovy” “visioneers” who prepared the way for Thiel, Musk, et al.^55^ The gist of that literature is that Silicon Valley has always been like this: the eugenics, misogyny, occultism, and toxic libertarianism (somehow entangled with a desire for authoritarian rule) were there at the beginning.^56^

Yet there have also always been other Silicon Valleys. The company most responsible for the Valley’s profit-sharing model, for instance, was Varian Associates – “associates” because the founders, Russell and Sigurd Varian, wanted their co-workers to see themselves as co-owners rather than employees. True, the Varian brothers grew up on a theosophist commune, which sounds pretty groovy and utopian; but that upbringing expressed itself mostly in the Varians’ left-liberal environmentalism, antifascism, and association with people who ran afoul of the House Un-American Activities Committee.^57^ They were creatures of the center-left whose consciences were pricked by their company’s contributions to nuclear weapons, yet who saw a well-armed democracy as necessary to check Russian influence and authoritarian rule – just the opposite of Sacks’ and Musk’s embrace of Russian authoritarianism today.

So, there’s always been a progressive Silicon Valley. There’s also always been a left Valley, exemplified by the Stanford students who conducted sit-ins and investigations to force the university to lessen its dependence on the military.^58^ Some of those people even shifted the national conversation about science and technology, much like – but counter to – what Musk et al. are doing today. One Stanford alum, Denis Hayes, was lead organizer of the first Earth Day in 1970 and later head of the Solar Energy Research Institute under Jimmy Carter; another, Stanton Glantz, was the antismoking activist who first exposed the “merchants of doubt” network of climate and cancer denialists.^59^

Those people represent an alternative, usable past to contrast with the extremist and frankly bizarre Silicon Valley lineage represented by Trump’s broligarchs. But so does the more numerous and boring Silicon Valley of the center-right. The companies that adopted the Varians’ profit-sharing template were mostly run by probusiness, virulently antiunion executives who had no objections to the military–industrial complex except when Pentagon bureaucracy hindered their ability to make money. A few of them were flamboyant showmen – Robert Noyce, Jerry Sanders, and Regis McKenna come to mind – whose boasts and hype paved the way for Steve Jobs and, eventually even for frauds such as Elizabeth Holmes and Sam Bankman-Fried.^60^ Yet the majority of Valley executives cultivated colorless and bland personae – “plain vanilla” as one, Charles Harwood of Signetics, described himself.^61^ Their king was Gordon Moore, the “quiet revolutionary” and unassuming foil to his frenetic Intel cofounders, Noyce and Andy Grove; and, yes, their politics was conservative, but in the old-fashioned sense of resisting change.^62^

Obviously, there are continuities between that Silicon Valley and today’s. Valley firms’ newsletters from the late sixties and early seventies were filled with male chauvinism, jingoism, and scorn for the antiwar and civil rights movements. The same companies were notorious for poisoning their employees and the surrounding groundwater. When employees and neighbors pushed back, Valley firms left for authoritarian regimes, mostly in Asia. When those regimes democratized – as in Portugal in 1974 and the Philippines in 1985 – Valley firms abandoned their plants rather than accommodate to democracy.

The big difference with today, though, is that the center-right Valley responded positively if begrudgingly to civil society’s demands. Corporate swimsuit competitions, for instance, ended in the mid-1970s, as women and people of color started moving up the corporate ladder. When South Korea and Taiwan democratized in the late 1980s and early 1990s, Valley firms stayed put – unlike in the Philippines and Portugal earlier. When the “Signetics Three” – Marta Rojas, Cathi Hee, and Cathy Bauerle – sued over exposure to toxic chemicals in their workplace, Signetics initially fought them, but later took the opportunity to become the leading champion of occupational health practices that are now the industry standard.^63^

Similar resistance followed by accommodation took place with respect to water pollution and microelectronics manufacturers’ collusion with organized crime. On each issue, coalitions of concerned citizens, journalists, and activists pushed Valley companies to reform: not always successfully, but not always unsuccessfully either. Even when they didn’t concede, Valley companies didn’t respond by wrecking the system like Musk is. Instead, they started to participate in democratic politics by forming political action groups and even lobbying for increased local taxes to pay for much-needed infrastructure.

In other words, Silicon Valley’s history is much more diverse than just the line of cranks and racists leading to Musk. Civil society, democratic give-and-take, sharing the wealth, the administrative state – these are Silicon Valley’s history, too. Recovering those histories undermines the broligarch’s narrative of heroic libertarian achievement.^64^ They and their predecessors achieved nothing without the help of the state, women, people of color, the left and center-right, and civil society more generally. Those other actors have not received as much historical attention, unfortunately – partly for reasons of prejudice, but also partly because some of them deliberately made themselves uninteresting. Which, in itself, is a choice worth defending – unlike the past decade of politics-as-reality-TV. So I say, let’s make Silicon Valley boring again!

## Executive Order 14168: Sex, gender, and common sense

**Ilana Löwy**

Centre de recherche médecine, science, santé et société, Paris, France

On the first day of his presidency, Donald J. Trump issued Executive Order (EO) 14168, which states:

My Administration will defend women’s rights and protect freedom of conscience by using clear and accurate language and policies that recognize women are biologically female and men are biologically male.

Sec. 2. Policy and Definitions. It is the policy of the United States to recognize two sexes, male and female. *These sexes are not changeable and are grounded in fundamental and incontrovertible reality*. Under my direction, the Executive Branch will enforce all sex-protective laws to promote this reality, and the following definitions shall govern all Executive interpretation of and application of Federal law and administration policy:

(a) “Sex” shall refer to an individual’s *immutable biological classification* as either male or female. . . .
(d) “Female” means a person belonging, *at conception*, to the sex that produces the large reproductive cell.
(e) “Male” means a person belonging, *at conception*, to the sex that produces the small reproductive cell.^65^

On March 4, 2025, in his Presidential Address to the joint session of Congress, President Trump reaffirmed the principle of dual humanity, although intriguingly he spoke of gender rather than biological sex:

I signed an order making the official policy of the United States government that there are only two genders, male and female. . . . What I have just described *is only a small fraction of the common sense revolution that is now, because of us, sweeping the entire world*. Common sense has become a common theme, and we will never go back.^66^

In the late twentieth and early twenty-first centuries, far-right organizations often contrasted the common sense of “the people” with the outrageous and unhinged views of the “woke” left and the arrogant dictates of the experts, imbued with their false certainties. The distrust of experts might have been amplified by the Covid pandemic.^67^ EO 14168 affirms the importance of the common sense understanding that humanity is composed of two biological sexes, male and female, that sexual identity is fixed at the moment of conception, and that it is defined by the size of the reproductive cells produced by a given individual. This definition mixes two different registers of knowledge: the duality of biological sex, known (presumably) since prehistoric times, and the notion, developed in the nineteenth century, that sex glands, testes and ovaries define biological sex.

Until the nineteenth century, human beings were classified at birth as males or females according to the form of their external genital organs. The development of anatomy and physiology, and in parallel observations made on individuals with intermediary sex traits – called hermaphrodites, then “intersex” persons, and today people with DSD (disorders of sexual development, or differences in sexual development) – undermined this vision. In the second half of the nineteenth century, the focus of sex definition switched from external sex organs to the presence of sex glands: testes and ovaries.^68^ In the early twentieth century, the definition of biological sex as grounded in the presence of sex glands was modulated by the description of the key role of sex hormones – estrogen, progesterone, and testosterone – in the development of secondary sexual traits.^69^ At that time, embryologists investigated in detail the development of male and female fetuses, while geneticists determined that, in mammals, females have two X chromosomes while males have one X and one Y chromosome. It became increasingly clear that the determination of sex during pregnancy is a multilevel process that implies several distinct mechanisms, which may align – or not.

Before the 1950s, physicians had limited ways to propose medical interventions to “intersex” children. This changed dramatically with the purification of sex hormones, the progress of plastic surgery, and the development of drugs such as cortisol. Experts, such as the Johns Hopkins pediatric endocrinologist Lawson Wilkins, increasingly proposed interventions to modify bodies of “intersex” children and make them conform more closely to the bodies of “normal” males or females. Wilkins wondered, however, what the psychological consequences of such interventions would be. For example, children with congenital adrenal hyperplasia had chromosomal formula XX, a uterus, and ovaries, but because of exposure to high levels of testosterone in the womb were born with male-looking external sexual organs and often also had a male body type. Before the 1950s, many of these children were raised as males because their doctors assumed that they would be happier living in a sex that corresponded to their visible anatomical traits. In the 1950s, these children were offered hormonal therapy and surgery that provided them with more female-like bodies. However, Wilkins was not sure whether these children would see themselves as girls or, because of their exposure to testosterone before birth, as boys. He hired the psychologist John Money to study the self-image of these children – a study that led to the coining of the term “gender,” meaning a person’s self-identity as a man or a woman.^70^

An especially dramatic case was that of people with Turner and Klinefelter syndromes. Women with Turner Syndrome (TS) do not have functional ovaries and are infertile. They do have characteristic body traits: short stature and less pronounced secondary sex characteristics. Men with Klinefelter Syndrome (KS) are also infertile, are tall, and often have a “feminine,” or rather intermediary body type. Nevertheless, children born with these conditions were classified at birth as female (TS) or male (KS) since their external sex organs looked perfectly “normal,” and were socialized accordingly as girls or boys. Their problems of sexual development, frequently observed at puberty, were attributed to hormonal perturbations.

Until the mid-1950s, technical problems hampered the direct visualization of human chromosomes. However, in 1949, Murray Barr, a neuroscientist, showed that all female mammal cells had a small inclusion (a dark spot) in the nucleus. The display of this inclusion, called a Barr body, provided a simple way of differentiating “chromosomal males” and “chromosomal females.” Lawson Wilkins invited Barr to study the “chromosomal sex” of patients in his clinics. To their great surprise, Barr found that TS individuals, who fully identified themselves as women, were “chromosomal males” and KS individuals, who fully identified themselves as men, were “chromosomal females.” KS and TS were redefined as extreme examples of “sex reversal.” Wilkins decided that, to protect their psychological well-being, people with these conditions should not be told that they were “nature’s freaks” and that their biological sex did not correspond to their gender.^71^

In 1956, thanks to the development of new technologies, cytogeneticists were finally able to visualize and study human chromosomes.^72^ In 1959, scientists found out that TS individuals have only one X chromosome (45X0), and KS individuals have two X chromosomes and one Y chromosome (47XXY).^73^ Since the Barr body was a marker of the presence of two X chromosomes, it was detected in KS but not TS individuals. People with these syndromes were not “nature’s freaks” and examples of “sex reversal” – just a telling illustration of the complexity of biological sex and the sex/gender nexus.

One of the key developments that marked the birth of modern science was the dissociation between common sense and the scientific world view. This dissociation was epitomized, historians and anthropologists have argued, by the Copernican Revolution.^74^ Copernicus’s magnum opus of 1543 affirmed that the common sense perception of the universe was mistaken.^75^ This view was initially limited to a small group of scholars. It took centuries of efforts – the diffusion of scientific theories, the training of specialists, the introduction of science into primary and secondary education, the translation and diffusion of textbooks, and the rise of institutions such as science museums that popularized science – to persuade the majority of people that Copernicus was right in this regard. In his Presidential Address, President Trump stated that “we are going to forge the freest, most advanced, most dynamic and *most dominant* civilization ever to exist on the face of this Earth.”^76^ One may conclude that this advanced, dominant civilization will propagate the common sense revolution. Welcome to the year 1542.

## “A dagger to the heart?” Climate change and the environmental sciences

**Thomas Zeller**

University of Maryland, College Park

Typically, historians study the past after a reasonable passage of time. The current administration’s onslaught on climate change and the environment has been so thorough, however, that it appears as if research in both areas occurred a very long time ago. Yet it has only been months since that research was axed from federal agencies. Even now there are deeper cuts on the horizon. A profound rupture is apparent. Support for research on global warming and the environmental sciences was a child of the Cold War. Now it is an orphan without a benefactor in the White House. Seen in a larger historical context, the current upheaval marks the end to over seven decades of extensive federal funding for the environmental sciences.^77^

This rupture (and all that has accompanied it) has, for some historians, called to mind authoritarian regimes as points of comparison, especially those of interwar Europe. As a group of Science, Technology, and Society (STS) scholars wrote in late February 2025, “we have been here before.”^78^ But there are differences.^79^ Those regimes did not go so far in dismantling science and its research institutions; in fact, they increased funding for projects deemed important for ideological or military goals. By contrast, the current administration has been sledgehammering the sciences. It aims to dramatically decrease the role of and spending for federal research agencies, including the Environmental Protection Agency (EPA), the National Science Foundation, and the National Oceanic Atmospheric Administration, all sites of research on climate change and the environment. The White House seeks not only to control *what* kind of knowledge is created, it also seeks to redefine *who* is creating knowledge and the regulations issuing from it. This erratic retreat will have massive domestic consequences not only for the United States but, given the extent of government funding and the hegemonic reach of American-funded science, the repercussions will be global.^80^ Based on the events of the last two months, this dramatic retreat and its chaotic implementation will amplify domestic tensions and accelerate the decline of the global role of the United States.

Historically speaking, the United States has been an important place for studying the environment, even while being the single biggest emitter of carbon dioxide. In early 2025, the federal government employed thousands of scientists and regulators producing environmental knowledge as well as regulations. While the current funding cuts appear to be indiscriminate in some scientific disciplines, a pattern has emerged for the environmental branches of the administrative state. At the EPA and the Department of Justice, employees working on environmental justice were some of the first to be fired or reassigned. Grants for environmental studies awarded by other federal agencies were canceled. Those in power are no longer interested in the differential impact of pollution and other kinds of environmental harm on poor communities and communities of color.

The current administration aims to extract environmental resources and reestablish the supremacy of fossil fuels. The overall mission of the EPA has changed dramatically. Rather than mitigating pollution and working for public health, the agency’s current administrator believes in “collaboration [with industry] rather than regulation.”^81^ Emission standards for industrial air pollutants and regulations for vehicle emissions face repeal or revision. Economic growth has become paramount; as a result, corporations will benefit. Scholars whose analytical toolbox includes the Capitalocene (a term used to designate the role of capitalism in climate change) will not be surprised to see a push to restore coal and oil as the main energy sources rather than renewable resources. Massive deregulation and dismantling of government had also been goals for Ronald Reagan in his first term in office. After a public outcry, he restored some of the former powers of the EPA after three years. The current president, however, appears to be hell-bent on defanging it. In the federal regulatory landscape of the United States, researching and setting new standards for clean water and air might revert back to states and municipalities, thus deepening the divide between states even more and in the most material of ways. A new form of federalism might emerge: states with higher levels of taxation on the coasts might decide to improve standards for clean air and water, while less affluent states might let them stagnate.

Two of the biggest casualties of the current purges are research and regulation regarding human-induced global warming. With a gleeful bit of Dracula-esque rhetoric, the current EPA administrator speaks of “driving a dagger through the heart of climate-change religion.”^82^ Research on climate at federal agencies has been halted and funding for such research at universities is in danger. The fear among researchers is palpable.^83^ While criticism of climate science is hardly new, an abrupt end to supporting it would be. Previously, various arms of the U.S. government have been some of the most important and consistent sponsors of climate science. The imprint of the Cold War is hard to overlook. Manipulating the weather for military purposes and creating computing capabilities to model large-scale climate forecasts are only two of the various aspects of government funding.^84^ Perhaps the most visible artifact of steady Cold War funding and long-term research is the Keeling Curve, which documents annual increases in atmospheric carbon dioxide measured at the Mauna Loa Observatory since 1958. One scientist called it the “most important environmental data set taken in the 20th century.”^85^ The current administration is considering cutting support for the observatory.^86^

The 2025 assaults threaten to rupture not only such longitudinal studies, but the entire infrastructure of climate science in the United States. Skeptics of global warming have long used the contested nature of knowledge production to disregard it.^87^ They used the fact that scientific findings are incomplete and preliminary as a pretext. It allowed them to claim that such knowledge is unreliable and uncertain. Now it is the future of climate research itself that is uncertain. The massive funding during and after the Cold War, which had undergirded it for decades, is appearing to ebb.

There is a paradox in recent developments. Despite its attacks on climate science and research, the current administration has ironically validated the reality of rising temperatures and sea levels through its expansionist desire to annex Greenland and Canada. Their northern reaches offer a geopolitically strategic Northwest Passage, a predicted result of climate change. Moreover, while many in the Global South will suffer as a result of climate change, both Greenland and Canada have the potential to benefit from longer growing seasons and, as a result, increased trade. The White House might turn a blind eye to climate science, but it seems eager to reap the regional benefits of global warming, which is a reality regardless of who occupies this seat of power.

One of the major takeaways from the topsy-turvy world of 2025 is that the environmental sciences are only now experiencing the end of the Cold War and its bountifulness. As historians have pointed out, the relationship between scientists and the state during this period was remarkably stable.^88^ It continued and even grew for another thirty-five years after the fall of communism in Eastern and Central Europe. What is so stunning about the current crisis is how hastily and suddenly political leaders are putting the brakes on science and technology. For several decades, working in the United States as a researcher or academic meant acquiring a position of relative privilege. Research institutes were cosmopolitan places, with European and other leaders worrying about brain drain. Currently, the blight of American science offers an opportunity for these locales to attract talent.

In the current chaotic moment, all bets are off. Uncertainty reigns and confusion diminishes the former global role of the United States in promoting the environmental sciences, especially as they relate to climate change. The Sputnik shock of 1957 solidified and expanded federal support for the sciences and for research on the environment; the Trump shock of 2025 seeks to end it.

## Science and ideology

**Mark Walker**

Union College, USA

The Nazi regime was clearly anti-intellectual, but although purges of Jewish and leftist scientists began in April of 1933, a little more than two months after Adolf Hitler was appointed chancellor, the institutional structures of science, including their funding, largely remained in place. These structures became more authoritarian, but were not transformed. There was a significant number of competent German scientists available and willing to move into the positions freed up by the firing of scholars for racial or political reasons and to work with the Nazi regime. By 1936 the institutional system of science had stabilized. There were some changes. Students or researchers who wanted entry into scientific institutions like universities had to fulfill racial and political criteria. Some disciplines or subjects were reduced or eliminated, while others were expanded. Scientists and physicians were enlisted to staff and run new programs for social engineering, like sterilization and the murder of the disabled, although the latter was not made public. But, by and large, most foreign scientists or émigrés who visited Germany at this time would have gotten the impression that German science had not changed much, except for the people who had left. Rearmament and war, especially after it began going badly for Germany, brought with it much greater change. Scientists, physicians, and engineers working in aerodynamics, pre-DNA genetics and heredity, or physical anthropology, for example, did not complain about a lack of support; indeed, there is evidence of such researchers receiving whatever they asked for. The German colonization of Poland and the western parts of the Soviet Union provided myriad opportunities for scientists, which were exploited. The more desperate the war for Germany became, the more many scientists, physicians, and engineers supported, participated in, or at least tolerated forced and slave labor, medical experiments with human subjects, and genocide.^89^

In the Soviet Union, Lenin slowed down radical reform when the New Economic Plan was introduced. The Soviet leadership recognized that it needed the help of “bourgeois specialists” to construct a communist state. Initially the most important scientific institution, the Academy of Sciences, was left largely alone, and most scientists who wanted to stay and work in the Soviet Union were able to do so. However, as in Nazi Germany, the universities were politicized early on, so that the educational system gradually began producing more and more scientists supportive of the new political order. In both countries a distinction was made between science at universities and in research institutes like those attached to the academy: scientists who did not wholeheartedly support the regimes were tolerated in research institutes if their work was seen as valuable; only scientists who were considered loyal were allowed to teach the next generation. After Lenin’s death, the transformation toward communism was accelerated by Josef Stalin. This period included forced collectivization and the infamous political purges, including of the military elite, the Communist Party itself, and also of science. The latter arguably culminated in the well-known growth of Lysenkoism and attacks on modern genetics. However, the limits of this politicization were also shown by the fact that, despite some initial efforts by politically active scientists, no such purges ever happened in physics. Thanks to the atomic bomb, this discipline was clearly valuable to the Soviet leadership.^90^

Although it is still very early in the second Trump administration, already enough damage has been done to science to make it tempting to compare the state of American science to how science fared under the Nazis and Soviets, perhaps the two most extreme examples we have of science being affected by ideology. In the short run, the Trump administration’s policies appear to be more chaotic and disruptive than in Germany or the Soviet Union. One gets the impression that civil servants were fired or coerced to resign and programs and policies were ended first and foremost simply because they could be through a ruthless application of the power of the executive branch. However, there does appear to be an anti-intellectual and therefore anti-scientific ideology at work, combined with an antagonism toward liberal social policies.

The obvious conclusion I would draw is that it will be better for science if the destructive policies of the Trump administration do not continue, and if scientists do not make their peace with the new order – as many did in Germany and the Soviet Union. I have studied examples of prominent scientists who were at first attacked, disrespected, and even threatened for ideological reasons. Because these scientists responded, not by condemning or rejecting the new political order, but instead by working hard to convince the ruling elites that both their scientific work and they themselves were worthy of support, their success ultimately led to their collaboration with and support of the ideological goals of the regime.

As someone who has studied the history of National Socialism for his entire professional career, including comparisons with other extreme political and ideological regimes, it is sobering to have to conclude that the current government of the United States is currently trending toward “fascism” with an apparent goal of becoming “totalitarian.” Scholars and other writers have often asked, why did Germans not do more to oppose the Nazis? When contemplating this myself, I have to recognize how I have responded to the actions of the second Trump administration. I have not quit my job, converted my savings and assets into cash, driven my family across the Canadian border, and applied for political asylum. I also have not moved to Washington, D.C. and used whatever assets I have to personally and physically protest outside the White House or Congress. But together with colleagues, I am at least writing this commentary to warn of the dangers of acquiescence to a regime headed in the direction of fascism.

## A regime change and the scientists’ response

**Mitchell G. Ash**

University of Vienna, Austria

Since the 1990s, when I witnessed first-hand the impact of the fall of communism on higher education and the sciences and humanities in East Germany, much of my scholarship has been concerned with the political history of science, defined as a relational history with multiple dimensions.^91^ Currently I am writing a book with the working title *Scientific Changes in Times of Political Upheaval*, which will consider how the interactions of scientific and humanistic research with political power were renegotiated during and following the major political regime changes of the twentieth century symbolized by the dates 1918, 1933/1938, 1945, and 1990. The potential relevance of this work to the situation now unfolding in the United States seems obvious. However, in order to understand what is happening, certain basic points must be stated clearly.

First: A genuine regime change is now underway in the United States, of which the attack on scientific institutions and knowledge is a part. Understanding the attack on the sciences and the humanities thus requires an analysis informed by political and constitutional history. In the past, U.S. presidents have attempted to extend the powers assigned to them in Article 2 of the U.S. Constitution in various ways, but never before has a president done so to the extent now being undertaken by Donald Trump and his enablers. The elimination of government agencies, arbitrary budget cuts, and dismissals of civil servants by an agency of questionable status headed by Elon Musk are being enacted by presidential fiat, without the consent or even consultation of Congress.^92^

Second: Calling this an attack on American democracy is fundamentally mistaken. Doing so presupposes that the term “democracy” includes both popular sovereignty and the rule of law; we are now learning that this is not true. Donald Trump and the Republican Party are misusing their marginal election victories, deliberately exaggerated in size and falsely termed a “mandate,” to legitimate an assault on the rule of law and the American state itself. This should have been obvious on Inauguration Day, January 20, 2025, when Trump issued an executive order (EO) ending birthright citizenship, thus abrogating unilaterally the Fourteenth Amendment to the U.S. Constitution, in order to enable the deportation of children of undocumented immigrants born in the United States. The sheer volume and variety of EOs, the speed with which they are being implemented, and the addition of ever new orders are deliberate tactics designed to weaken, confuse, and distract opposition. Former Trump advisor Steve Bannon called this “flooding the zone” in 2016. Now a tactic designed to confuse the media has been repurposed to achieve regime change.

Opposition to all this comes mainly from the courts at present. Multiple lawsuits have been filed against EOs with a high success rate. Unfortunately, the confusing variety of cases and parties involved and the lack of coordination among them directly results from the “flood the zone” tactics, compounded by the fragmented patchwork of single-issue civil society groups pursuing litigation alongside state governments. It was unclear at first whether Trump and Musk would try to evade or even violate court orders, and thus take the final step to authoritarian rule. In the case of alleged Venezuelan gang members deported to El Salvador without a hearing, many of whom have not been charged with crimes in the United States other than entering the country illegally, Trump and Musk have since violated a court order to return them, but are also moving to appeal this and other negative court rulings, hoping perhaps that the Supreme Court’s reactionary majority will rule in their favor on the basis of a doctrine popular in right-wing circles called the “unitary executive,” thus giving their actions a semblance of legality. An earlier ruling by the court in a case ironically named “Trump v. United States” grants Trump himself absolute immunity from prosecution for vaguely defined “official acts,” even if these are blatantly illegal.^93^ Massive public protests began in March; by April they were coordinated across the United States.^94^ Whether the Democratic Party can oppose Trump and his enablers effectively is also unclear. At present the party’s establishment appears to be focused on winning the mid-term elections in 2026, oblivious to the risk that regime change might already be complete, and free elections impossible by then.^95^

Comparisons offered by historians and others with the speed of the Nazi takeover of power in 1933 remind us how quickly democracies can be dismantled.^96^ Ultimately, however, comparisons of this kind establish differences rather than similarities. The political and economic situation of Germany in 1933 was fundamentally different from that of the United States today, and the mix of supine acceptance of and active support for Trump’s onslaught by the Republicans in Congress also differs from the outright collapse and subsequent abolition of political parties in that year.

What the destruction now taking place in American research institutions has in common with past regime changes is that it is not an act of science policy, but rather a subset of the destruction of the American state itself. The threat has both ideological and institutional dimensions. Clearly ideological are the attacks on so-called wokeness, diversity policies and alleged antisemitism in the social sciences and humanities, exemplified most recently by an EO authorizing Vice President JD Vance to cleanse the Smithsonian Institution of supposedly false and “divisive” exhibits and “restore truth and sanity to American history.”^97^ Vance sits on the Smithsonian board, but has no legal right to carry out such censorship.

The institutional dimension is exemplified by the withholding of $400 million in already approved federal grants from Columbia University as punishment for alleged “failures” to protect Jewish students from violent demonstrators. The use of grant withholding as blackmail shows that members of STEM disciplines who might think that they are safe from ideological attacks are deeply mistaken. This is most obvious in medical research, given the appointments of Robert F. Kennedy, Jr. as Secretary of Health and Human Services and of Stanford University professor Jayanta Battacharya as the director of the National Institutes of Health, the largest funder of biomedical science in the United States. Both have advocated debunked positions on the impact of vaccines and public health measures against the Corona virus.^98^ Going beyond biomedical science, Lee Zeldin, administrator of the Environmental Protection Agency, has announced sweeping “deregulation” of the fossil fuel industries, saying that “We are driving a dagger straight into the heart of the climate change religion.”^99^ Severe cuts to the National Oceanic and Atmospheric Administration, which provides data needed for accurate weather prediction, show that technical institutions that provide clear economic benefits in return for government support are no better off than history or biomedicine.^100^ In some cases, attempts are being made to rehire dismissed personnel as the relevance of their work becomes clear, but the damage has been done.

How can people working in the sciences fight back? Discussions thus far rely on the conventional narrative of the sciences and scholarship as innocent victims of power politics. My own work challenges this narrative. Since organized science and scholarship have had power structures of their own for more than a century, it would be more accurate to speak of an unequal struggle among two power blocs.^101^ In the case of Columbia University the regime successfully pursued a tactic of divide and rule, utilizing accusations that the institution had responded insufficiently to antisemitism to bring universities to heel one at a time, as is also being done with large law firms. In mid-April, however, the president of Harvard University resisted administration demands for reforms in governance, hiring, and admissions, as well as an external audit of multiple academic programs accused of advancing antisemitism, arguing that such demands exceeded the authority of the federal government: “The university will not surrender its independence or relinquish its constitutional rights.”^102^ In response, the administration froze $2.5 billion in federal funds that had been awarded to the university.^103^ The dispute is ongoing; whether other universities will fall into line remains to be seen.

Active resistance came at first from graduate students and postdocs who supported the initiative “Stand Up for Science” in early March.^104^ Leaders such as Michael S. Roth, president of Wesleyan University and former president of the American Association of University Professors, continue to speak out for academic freedom,^105^ and disciplinary bodies such as the American Historical Association have taken similar stands, most recently against the attack on the Smithsonian Institution.^106^ The leadership of the American Association for the Advancement of Science (AAAS) and the editor of the AAAS journal *Science*, H. Holden Thorp, published strong statements at the beginning of the Trump regime.^107^ Sadly, the leadership of the National Academies of Sciences (NAS) has not done so thus far. NAS President Marcia McNutt stated in an interview that she does not think that public statements have any impact on policy, and prefers working with responsible government officials, without saying whom she could possibly mean.^108^ In strong contrast to this view, more than 1,900 members of NAS published a protest letter at the end of March stating that “We hold diverse political beliefs, but we are united as researchers in wanting to protect independent scientific inquiry. . . . the nation’s scientific enterprise is being decimated.”^109^

The long-standing arrangement in which academic science received autonomy and federal funding in exchange for producing useful knowledge is indeed threatened. References to the usefulness of the knowledge provided by government agencies or research institutions may not be persuasive when rationality has lost its standing and raw power rules. Elon Musk’s response to protests from Republican representatives about problems caused by budget cuts in their districts reinforces this impression; he acknowledged that mistakes are possible, gave them his mobile phone number, and said they could call him if they had any problems. He has yet to do this for Democrats.^110^

High-level initiatives to recruit top scientists by European institutions have begun, but whether a coherent European strategy in this direction can be developed and implemented quickly or whether such efforts will matter remain to be seen.^111^ The disruptive effects of the Trump regime’s measures on international research networks clearly benefit China, which already leads the world in scientific publications. Yet again we are learning, as my own work and that of other scholars on the sciences in Nazi Germany and the Soviet Union have shown, that excellent science can be produced in dictatorships when the work supports military or political projects. The comforting mantra that democracy itself somehow automatically guarantees the autonomy of science and scholarship will need to be reconsidered, if the world’s predominant scientific nation becomes an autocracy.

## Question obedience: Stand up for public science and public knowledge

**Donna Haraway**

University of California, Santa Cruz, USA

On March 7, 2025, in solidarity with national Stand Up for Science actions, I and several colleagues from the Humanities Division, the Physical and Biological Sciences Division, and the Science and Justice Research Center at the University of California Santa Cruz organized large, enthusiastic gatherings on both the Coastal Science Campus and the main campus in front of the science library.^112^ We are following up with monthly meetings to share information and strategy and to work collectively for the sciences and the science and justice we love and need. Our call is for strong alliances across fields for free speech, university self-government, academic freedom, and public research. We recognize that young students and researchers – from undergrads losing scholarships that allow them to be the first members of their families to attend college, through postdocs and assistant professors – are already the most profoundly damaged, with more wreckage promised.

This local organizing is a small thing in the larger crisis, but it signifies and enacts two critical things: 1) we will not obey orders to destroy our people and our sciences or other knowledge projects or submit to authoritarian, indeed fascist, destruction of the apparatuses for crafting and nurturing public research and teaching, whether those orders come from the federal government or from our own universities; and 2) we will think and act together.^113^ Never have collective thought and action been more necessary. Capitulating to authoritarianism breeds more authoritarianism and protects nobody and nothing, no matter the cowardly illusion that obeying will make the tyrants focus their attention elsewhere.

I offer a terrible comparison to make my point. Perhaps we are not quite at the point the world-renowned existentialist philosopher Martin Heidegger faced of full surrender of his university to the Nazis, whatever his actual views. But we are closer than many wish to admit. Prompted by the current crisis in the university, I carefully read Heidegger’s 1933 capitulation to the Nazification of the German university in his Rectorship Address on “The Self-Assertion of the German University,” accepting his appointment as Rector under the Nazis.^114^ I read this doleful text alongside Columbia University’s Interim President Katrina Armstrong’s and Columbia’s trustees’ groveling capitulation to the current authoritarian regime in the United States over charges of unaddressed antisemitism at Columbia.^115^ I use the term “Trump regime” instead of “Trump administration” to signal its abundant use of illegal and unconstitutional means.

In the current regime, criticism of Israel or of Zionism and support of Palestine are easily equated to antisemitism, and such protest in any form is more and more harshly punished in our universities.^116^ Columbia’s severe, ongoing punishments of its student protestors after pro-Palestinian protests in spring 2024 counted for nothing. Trump and his Christian nationalist and other cronies, in Israel and in the United States, want more. They want to bring universities to their knees, and they are succeeding. Public sciences and science and justice research and teaching are principal targets. Both the 1933 and 2025 documents are full of cowardice and fear of the tyrant. Neither Heidegger nor Columbia’s highest officials proposed any resistance to growing authoritarianism and fascism. In philosophical language that betrays philosophy, both academic leaders appealed to the highest values of Western civilization (freedom, science, value of free expression, commitment to community) in speech acts that chill the soul. Further, the “thought” behind both Heidegger’s infamous speech and Columbia’s capitulation depends on human exceptionalism. This is a root of both German exceptionalism in the 1930s and U.S. exceptionalism now. The language of cowardice and exceptionalism is numbing: “never before in history,” “worth any sacrifice,” “highest values of humanity,” and so forth.

Liberty, science, and humanity deserve better.

It is impossible to miss the terrible history and terrible irony that both Heidegger’s and Columbia’s statements exist in a world that abundantly continues to hate and all-too-frequently kill Jews. Opposing antisemitism in its constantly reinvented forms remains urgent, as do support of the Palestinian people and opposing Islamophobia. Threatening independent research and public science for climate, health, biodiversity, and more is thinly disguised by Orwellian pursuits of antisemitism. Holding together Israelis and Palestinians, Jews and Arabs, is what requires actual “thought” (thinking in Hannah Arendt’s sense), and we are nowhere near able to do that, if our current mess is any evidence.

Columbia could have litigated the constitutionally dubious use of Title VI of the Civil Rights Act in the federal bullying and extortion.^117^ It did not. Specifically, Columbia University’s capitulation included:

**“Placing academic departments under government-mandated receivership** – effectively stripping faculty of control over curriculum and research agendas.

**Expanding surveillance, policing, and arrests** of student activists, with an explicit focus on suppressing political protest.

**Replacing a faculty, staff and student-driven disciplinary panel** with the executive mandate of the university president.

**Forcing the adoption of a definition of antisemitism** that equates criticism of the Israeli government with discrimination – contradicting established First Amendment protections.”^118^

It is not surprising that research and the sciences are at the center of these matters. Major research universities are between the devil and the deep blue sea due to a Faustian bargain embedded in post-World War II U.S. scientific organization and funding, with universities’ reliance on immense federal support, significantly enabled by U.S. global hegemony and the role of science and technology in the Cold War. This was the foundation of public science, including my own science PhD. Progressively, funding of the immensely expensive research apparatus has depended also on large corporate investment, privatizing for profit what should be public knowledge. Large universities are large corporations. Both the public and private streams of funding, the economic interests and politics of major donors, the progressively distant relation to research and teaching among trustees and higher administrators, and the weakening of faculty and student governance have all set up conditions for the perfect storm of capitulation to authoritarianism now raging. Defying dubiously legal federal orders backed by the threat of defunding the research apparatus itself, with implications for every field of knowledge in the university, takes both individual and institutional courage and a united front of institutions and people.

The charge of unaddressed antisemitism according to Article VI of the Civil Rights Act that was used to extort concessions from Columbia is not the main mechanism being used to cut federal grants, defund programs, and obliterate whole departments of research and teaching across divisions and fields. But more than sixty universities have been threatened for investigation under this guise. Instilling fear is the point. Fear solicits obedience. Continuing to have any hint of diversity, equity and inclusion programs is another favorite hook used by the Trump regime for defunding, threatening to refuse to hire graduates of noncompliant schools or programs (e.g., Georgetown Law School), or otherwise damaging research and teaching.^119^ Pausing grant application reviews; canceling necessary federal meetings; eliminating staff personnel; blocking payments; canceling scholarships for underserved student populations; canceling or denying grants that have any hint of addressing climate change, biodiversity, or environmental injustice: all these mechanisms and more are in play. So that they can protect their research, postdocs, students, and lab staff – scientists whom I know – eliminate words like “climate” or “biodiversity” in an effort to slip grant proposals past the Trump regime’s thought police. About one-third of the budget at my school, the University of California, comes from federal funds, much of which is threatened. Finding ways to support programs and people in the midst of this is, to say the least, a major challenge.

Columbia University is not uniquely to blame for capitulating. Very few upper administrators, much less trustees and governing boards, have made strong statements affirming bedrock principles of free speech, academic freedom, and university self-government, including the responsibility for discipline where warranted. Much less have they called for and organized a collective, across-the-nation, unified refusal to obey rogue federal orders. Where are the highest officers of colleges and universities, when the obligation has never been more urgent to nurture practical action for creative, effective, inclusive knowledge-making in labs, classrooms, and public spaces on our campuses, whether that knowledge is popular or not?^120^

Sciences for earthly flourishing cannot grow in such cowardly worlds. It is past time to return to our legacy of powerful collective movements and serious science for the people, this time for both human and more than human beings. Remembering these movements gives us heart – and ideas. There are too many places to begin. I could start in the European seventeenth century, with its astonishing invention of material, social, and literary technologies for establishing matters of fact that are not beholden to church or state.^121^ I could begin in the 1920s in England with progressive labor movements and proscience histories like *Science at the Crossroads*.^122^

But I want to begin closer to home, with the proscience, antiwar left and feminist movements of the 1970s and since. I can only list; there is no space to narrate and analyze. As anchors, I remember the Emergency Committee of Atomic Scientists, founded by Albert Einstein and Léo Szilárd in 1946, and the Union of Concerned Scientists, formed in 1968 at MIT to “devise means for turning research applications away from the present emphasis on military technology toward the solution of pressing environmental and social problems.”^123^ I was a graduate student in biology at Yale in 1968, and we biology graduate students were organizing – as scientists – against racism, sexism, and chemical and biological warfare. In 1971, women in my cohort at Yale and other junior cell and developmental biologists founded Women in Cell Biology to fight against sexism in science and for a robust and inclusive molecular, cell, and developmental biology.^124^

The Boston Women’s Health Collective; field-making feminist history of science and science studies panels in the 1970s organized by Sandra Harding at the National Women’s Studies Association; the *Radical Science Journal*; Science for the People as organization and publication; the Science and Justice Research Center organized at my university by Jenny Reardon; the antinuclear direct action movement; Evelyn Hammond’s organizing, research, teaching, and database curating for women of color in science; the Defend Science Movement launched in 2005 to counter antiscience in the George W. Bush presidency; environmental and climate science and activism engaging publics of myriad kinds; the Indigenous Action Task Force in Earth sciences; and individuals and groups engaged in saving data and websites from ideological erasure. This pointillist list in tangled time webs only hints at what we have to draw from for reconceptualizing and organizing our urgent struggle against Earth-and-earthling-destroying authoritarianism.

I end with ties connecting us as Americans, North and South Americans, that is. Resistance to authoritarianism and fascism in knowledge-crafting and nurturing is a global struggle. I end also in memory of my friend and colleague Sandra Harding, who died on March 5, 2025. Sandra proposed what she called “strong objectivity” to ground research in less biased, more capacious, less masculinist, racist, capitalist, and “Western” scientific practices.^125^ Her goal was to strengthen standards for what can count as that precious thing we call objective science. Ongoing renewal and revision of material, literary, and social technologies for establishing matters of fact are necessary for more adequate objectivity, for situated knowledges that are resistant to authoritarianism and other abuses.^126^ Trump’s and other antiscience regimes draw strength from breakdowns in public-facing social and literary technologies and the hypertrophy of destructive material technologies.

Harding continued for decades to reach beyond parochial epistemologies and practices. In later years, she collaborated with Latin American scholars to build “decolonial” sciences and science studies.^127^ My favorite fruit of that work is the journal *Tapuya: Latin American Science, Technology and Society*, beginning with a gathering in 2016 committed to bringing Latin American work and perspectives to a global audience. Nonparochial feminist science studies shaped the whole project. Scholars like Tania Pérez-Bustos of the Universidad Nacional de Colombia, Vivette García Deister of the Universidad Nacional Autónoma de México, Leandro Rodriguez-Medina of the Universidad Alberto Hurtado in Chile, Tiago Ribeiro Duarte of the Universidade de Brasília, and many more make the project flourish. The name “Tapuya” derives from the Quechua word “tapuy,” meaning “to question.” Always vulnerable but also fiercely powerful, knowledge-making is a questioning process. These are the kinds of knowledge-making that authoritarians and fascists especially hate. We in the United States are not alone, and we will not obey.

Sciences for Earthly Survival!

Run Fast, Bite Hard!

## Notes

1. Project 2025 Presidential Transition Project. *Mandate for Leadership: The Conservative Promise* (The Heritage Foundation, 2023), <https://static.project2025.org/2025_MandateForLeadership_FULL.pdf> (18 April 2025); Robert Reich, “Trump’s Playbook is Viktor Orbán’s,” 15 April 2025, <https://substack.com/home/post/p-161243190> (18 April 2025). Orbán also pursued a policy of controlling scientific research in Hungary. Phys.org, “Hungary Passes Controversial Science Sector Reform Bill,” 2 July 2019, <https://phys.org/news/2019-07-hungary-controversial-science-sector-reform.html> (18 April 2025).

2. Quoted in Molly Redden, Andy Kroll, and Nick Surgey, “‘Put Them in Trauma’: Inside a Key MAGA Leader’s Plans for a New Trump Agenda,” *Propublica*, 28 October 2024, <https://www.govexec.com/management/2024/10/inside-key-maga-leaders-plans-new-trump-agenda/400607/> (18 April 2025).

3. A useful chart of federal obligations since 1951 for research and development expressed in 2017 dollars, but one that requires deep contextual interpretation, may be found at: “A Brief Annotated History of Federal R&D Obligations: FYs 1951–2024,” 2025, <https://ncses.nsf.gov/pubs/nsf25332> (1 April 2025).

4. Federal Register, “2025 Donald J. Trump Executive Orders,” 2025, <https://www.federalregister.gov/presidential-documents/executive-orders/donald-trump/2025> (18 April 2025).

5. “Defending Women from Gender Ideology Extremism and Restoring Biological Truth to the Federal Government [Executive Order 14168],” 30 January 2025, <https://www.federalregister.gov/documents/2025/01/30/2025-02090/defending-women-from-gender-ideology-extremism-and-restoring-biological-truth-to-the-federal> (18 April 2025).

6. Joshua P. Cohen, “Hydroxychloroquine, a Drug Trump Promoted to Treat Covid-19, Linked to 17,000 Deaths, Estimates Show,” *Forbes*, 7 January 2024, <https://www.forbes.com/sites/joshuacohen/2024/01/07/trump-promoted-hydroxychloroquine-to-treat-covid-19-a-drug-now-linked-to-17000-deaths/> (1 April 2025).

7. “Ending Illegal Discrimination and Restoring Merit-Based Opportunity [Executive Order 14173],” 31 January 2025, <https://www.federalregister.gov/documents/2025/01/31/2025-02097/ending-illegal-discrimination-and-restoring-merit-based-opportunity> (18 April 2025).

8. “President’s Council of Advisors on Science and Technology [Executive Order 14177],” 31 January 2025, <https://www.federalregister.gov/documents/2025/01/31/2025-02121/presidents-council-of-advisors-on-science-and-technology> (18 April 2025).

9. Rene Marsh and Ella Nilsen, “Trump Officials Fired Nuclear Staff Not Realizing They Oversee the Country’s Weapons Stockpile, Sources Say,” *CNN*, 14 February 2025, <https://www.cnn.com/2025/02/14/climate/nuclear-nnsa-firings-trump/index.html> (18 April 2025); Tara Copp and Anthony Izaguirre, “Trump Administration Tries to Bring Back Fired Nuclear Weapons Workers in DOGE Reversal,” *AP*, 16 February 2025, <https://apnews.com/article/nuclear-doge-firings-trump-federal-916e6819104f04f44c345b7dde4904d5> (18 April 2025); Sharon LaFraniere, Minho Kim, and Julie Tate, “DOGE Cuts Reach Key Nuclear Scientists, Bomb Engineers and Safety Experts,” *The New York Times*, 17 March 2025, <https://www.nytimes.com/2025/03/17/us/politics/federal-job-cuts-nuclear-bomb-engineers-scientists.html> (18 April 2025).

10. Adam Cancryn and Erin Schumacher, “Fauci Allies, Covid Vaccine Officials Get Ax at NIH,” *Politico*, 2 April 2025, <https://www.politico.com/news/2025/04/02/fauci-allies-covid-vaccine-officials-dismissed-nih-00265714> (18 April 2025); Kerry Pickett, “Wife of Dr. Anthony Fauci Reassigned from NIH to Indian Health Service’s Regional Posts,” *The Washington Times*, 2 April 2025, <https://www.washingtontimes.com/news/2025/apr/2/christine-grady-anthony-faucis-wife-reassigned-nih-indian-health/> (18 April 2025).

11. Jeff Nesbit, “Trump and Kennedy Just Put America at Risk,” *The Contrarian*, 1 April 2025, <https://contrarian.substack.com/p/trump-and-kennedy-just-put-america> (1 April 2025). The Associated Press, “Here’s Where Jobs and Programs Are Being Cut at the Nation’s Top Health Agencies,” *AP*, 1 April 2025, <https://apnews.com/article/trump-hhs-cdc-fda-nih-cms-layoffs-5aba829b829d9e1a0167c4a0d968> (10 April 2025).

12. Leah Douglas and Marisa Taylor, “Trump Administration Starts Mass Layoffs at Health Agencies,” *USA Today*, 1 April 2025, <https://www.usatoday.com/story/news/politics/2025/04/01/trump-mass-layoffs-cdc-da/82754162007/> (18 April 2021); Jared S. Hopkins, “Drug Development is Slowing Down After Cuts at FDA,” *The Wall Street Journal*, 17 April 2025, <https://www.wsj.com/health/healthcare/drug-development-is-slowing-down-after-cuts-at-the-fda-f22369cf> (17 April 2025); Sophie Gardner, “Which Jobs Were Cut at CDC? Here’s a List,” *Politico*, 3 April 2025, <https://www.politico.com/news/2025/04/03/which-jobs-were-cut-at-cdc-heres-a-list-00271509> (10 April 2025).

13. Nick Evans, “Save the Minerva Research Initiative – Again,” *Breaking Defense*, 11 April 2025, <https://breakingdefense.com/2025/04/save-the-minerva-research-initiative-again/> (18 April 2025).

14. Aishvayra Kavi and Edward Wong, “ Workers Forced to Leave Foreign Policy Center as Trump Presses Shutdown,” The New York Times, 3 April 2025, (17 April 2025).

15. Eric Berger, “Trump White House Budget Proposal Eviscerates Science Funding at NASA,” *Arstechnica*, 11 April 2025, <https://arstechnica.com/space/2025/04/trump-white-house-budget-proposal-eviscerates-science-funding-at-nasa/> (18 April 2025); Sandra Erwin, “Intelligence Agency Copes with Workforce Reductions Amid AI Modernization,” *Space News*, 10 March 2025, <https://spacenews.com/intelligence-agency-copes-with-workforce-reductions-amid-ai-modernization/> (1 April 2025); Kathryn M. Olesko, “The Politics of Standards,” *Science in America*, 27 February 2025, <https://kathrynolesko.substack.com/p/the-politics-of-standards> (1 April 2025); Alejandra Burunda, “ Major Budget Cuts Proposed for the National Oceanic and Atmospheric Administration,” *NPR*, 11 April 2025, <https://www.npr.org/2025/04/11/nx-s1-5361366/major-budget-cuts-proposed-for-the-national-oceanic-and-atmospheric-administration> (18 April 2025); Jeffrey Mervis, “NSF Scraps Most Outside Advisory Panels,” *Science*, 15 April 2025, <https://www.science.org/content/article/nsf-scraps-most-outside-advisory-panels> (16 April 2025); Jeffrey Mervis, “NSF Halts Grants Awards While Staff Do Second Review,” *Science*, 16 April 2025, <https://www.science.org/content/article/nsf-halts-grant-awards-while-staff-do-second-review> (17 April 2025); Jeffrey Mervis, “NSF Starts to Kill Grants That Violate Trump’s War on Diversity Efforts,” *Science*, 18 April 2025, <https://www.science.org/content/article/nsf-starts-kill-grants-violate-trump-s-war-diversity-efforts> (18 April 2025); Erik Stokstad, “Trump Swings Budget Axe at USGS Biology Research,” *Science*, 18 April 2025, <https://www.science.org/content/article/trump-swings-budget-ax-usgs-biology-research> (18 April 2025).

16. Gisèle Muller-Parker, Susan Brennan, and Erik C. Jones, “Cancel Cuts to Graduate Research Fellowships,” *Science* 367 (2020): 519: Dan Garisto, “NSF Slashes Prestigious PhD Fellowship Awards by Half,” *Nature*, 10 April 2025, <https://www.nature.com/articles/d41586-025-01098-9> (18 August 2025); Dan Garisto, “US Universities Curtail PhD Admissions Amid Trump Science Funding Cuts,” *Nature*, 27 February 2025, <https://www.nature.com/articles/d41586-025-00608-z> (18 April 2025).

17. Tony Romm, “Donald Trump Crippled U.S. Technology and Science Policy,” *Vox*, 31 March 2017, <https://www.vox.com/2017/3/31/15139966/trump-white-house-technology-science-policy> (1 April 2025); Jacob Carter, Gretchen Goldman, and Charise Johnson, “Science Under Trump: Voices of Scientists at 16 Federal Agencies” [Union of Concerned Scientists], 7 August 2018, <www.ucsusa.org/2018survey> (1 April 2025); Jacob Carter, Gretchen Goldman, Genna Reed, Peter Hansel, and Michael Hapern, “Sidelining Science Since Day One,” *Union of Concerned Scientists*, 13 July 2017, <https://www.ucs.org/resources/sidelining-science-day-one> (18 April 2025); Dan Diamond, “Trump Officials Interfered with CDC Reports on Covid-19,” *Politico*, 11 September 2020, <https://www.politico.com/news/2020/09/11/exclusive-trump-officials-interfered-with-cdc-reports-on-covid-19-412809> (1 April 2025).

18. U.S. Government Accountability Office, *Operation Warp Speed* (GAO-21-319) (GAO, 2021), <https://www.gao.gov/assets/gao-21-319.pdf> (1 April 2025).

19. Alice Callahan, “Leading Nutrition Scientist Departs N.I.H., Citing Censorship,” *The New York Times*, 16 April 2025, <https://www.nytimes.com/2025/04/16/well/kevin-hall-nutrition-retirement-nih-censorship-rfk-maha.html> (18 April 2025).

20. Teddy Rosenbluth and Rebecca Robbins, “Trump-Allied Prosecutor Sends Letters to Medical Journals Alleging Bias,” *The New York Times*, 18 April 2025, <https://www.nytimes.com/2025/04/18/health/trump-martin-us-attorney-medical-journals.html> (18 April 2025). I thank Lissa Roberts for this reference.

21. Makiya Seminera, “A Look at the Universities with Federal Funding Targeted by the Trump Administration,” *AP*, 15 April 2025, <https://apnews.com/article/harvard-trump-federal-cuts-universities-protests-8fa92331b2780394ea171b0b32d5d243> (16 April 2025); Liz Essley Whyte, “The Little-Known Bureaucrats Tearing Through American Universities,” *The Wall Street Journal*, 16 April 2025, p.A1, A10. Some historians have argued that the administration is using antisemitism on campuses as a pretext for the intervention into higher education. Christopher Browning, “Trump, Antisemitism, and Academia,” *The New York Review of Books* 72, no. 6, 10 April 2025, p.42.

22. Science News Staff, “Surprise! White House Asks for ‘Permanent’ Block on NIH’s Sudden Indirect Cost Cap,” *Science*, 4 April 2025, <https://www.science.org/content/article/indirect-cost-surprise-nih-lawsuits-kennedys-mistakes-trump-tracker> (18 April 2025); Robert M. Rosenzweig, “The Politics of Indirect Costs,” *Council on Governmental Relations. 50th Anniversary, 1948–1998. Journal of Papers* (Council on Governmental Relations, 1998), pp.1–12, <https://www.cogr.edu/sites/default/files/A_Continuing_Evolution_-_Responding_to_Federal_Requirements.pdf> (18 April 2025).

23. Nicholas Riccardi, “Trump and Musk Aren’t the First to Make Deep Cuts. Clinton-era Reinventing Government Saved Billions,” *AP*, 23 February 2025, <https://apnews.com/article/trump-musk-doge-clinton-reinventing-government-gore-a95795eb75cacc03734ef0065c1b0a6d> (15 March 2025); Benjamin Siegel and Alexandra Hutzler, “Musk Compares DOGE to Clinton’s Government Efficiency Initiative, But There Are Big Differences,” *AOL Today*, 19 February 2025, <https://www.aol.com/musk-compares-doge-clintons-government-000046914.html> (25 March 2025).

24. “A Brief Annotated History of Federal R&D Obligations: FYs 1951–2024,” 10 April 2025, <https://ncses.nsf.gov/pubs/nsf25332> (April 18, 2025).

25. Frank Damurad, “1933 May Be Closer than We Think,” *History News Network*, 8 September 2019, <https://www.historynewsnetwork.org/article/1933-may-be-closer-than-we-think> (5 April 2025).

26. Mark Jones, “Will 2024 Be the New 1933?,” *Project Syndicate*, 29 January 2024, <https://www.project-syndicate.org/commentary/conservative-complicity-liberal-naivety-could-help-far-right-to-power-by-mark-jones-5-2024-01> (5 April 2024); Gerard Baker, “America Isn’t Nazi Germany, but It Looks a Lot Like 1933,” *The Wall Street Journal*, 18 March 2024, <https://www.wsj.com/opinion/america-isnt-nazi-germany-but-it-looks-a-little-like-1933-3f99b081> (5 April 2024).

27. Neel V. Patel, “America’s Brightest Minds Will Walk Away,” *The New York Times*, 3 April 2020, <https://www.nytimes.com/2025/04/03/opinion/young-american-scientists.html> (5 April 2025).

28. Stefan Raff-Heinen and Fiona E. Murray, “Don’t Quit the Long Game,” *Science* 388 (2025): 9; Alexandra Witze, “75% of US Scientists Who Answered *Nature* Poll Consider Leaving,” *Nature* 640 (2025): 299–300.

29. Catherine Offord, “Overseas Universities See Opportunity in U.S. ‘Brain Drain’,” *Science*, 17 March 2025, <https://www.science.org/content/article/overseas-universities-see-opportunity-u-s-brain-drain> (5 April 2025).

30. Quoted in Michael Gardner, “Max Planck Society Offers a Haven for Top US Researchers,” *University World News*, 21 February 2025, <https://www.universityworldnews.com/post.php?story=20250221121216853> (18 April 2025).

31. Quoted in Alexander Freund, “Dear U.S. Researchers: Welcome to Germany!,” *Deutsche Welle*, 27 March 2025, <https://www.dw.com/en/dear-us-researchers-welcome-to-germany/a-72058292> (18 April 2025).

32. Lisa Friedman, “Depleted Under Trump, a ‘Traumatized’ E.P.A. Struggles With its Mission,” *The New York Times*, 23 January 2023, <https://www.nytimes.com/2023/01/23/climate/environmental-protection-agency-epa-funding.html> (18 April 2025).

33. Donald MacKenzie and Graham Spinardi, “Tacit Knowledge, Weapons Design, and the Uninvention of Nuclear Weapons,” *American Journal of Sociology* 101 (1995): 44–99.

34. Sharon LaFraniere, Minho Kim, and Julie Tate, “DOGE Cuts Reach Key Nuclear Scientists, Bomb Engineers, and Safety Experts,” *The New York Times*, 17 March 2025, <https://www.nytimes.com/2025/03/17/us/politics/federal-job-cuts-nuclear-bomb-engineers-scientists.html> (1 April 2025).

35. Yusuf Khan, “Shortfall in Young Engineers Threatens Nuclear Renaissance,” *The Wall Street Journal*, 11 September 2024, <https://www.wsj.com/articles/shortfall-in-young-engineers-threatens-nuclear-renaissance-b63c6642> (1 April 2025).

36. Office of Nuclear Energy, “U.S. Sets Targets to Triple Nuclear Energy Capacity by 2050,” U.S. Department of Energy, 12 November 2024, <https://www.energy.gov/ne/articles/us-sets-targets-triple-nuclear-energy-capacity-2050> (1 April 2025).

37. Ulrich Beck, *Risk Society: Towards a New Modernity*, trans. Mark Ritter (London: Sage, 1992), p.21.

38. Lindsay M. Krall, Allison M. Macfarlane, and Rodney C. Ewing, “Nuclear Waste from Small Modular Reactors,” *Proceedings of the National Academy of Sciences* 119, no. 23 (2022): e2111833119.

39. Emma Derr, “Energy Studies and Models Show Advanced Nuclear as the Backbone of Our Carbon-Free Future,” *Nuclear Energy Institute*, 29 July 2022, <https://www.nei.org/news/2022/studies-and-models-show-demand-for-adv-nuclear> (1 April 2025).

40. Nuclear Weapons, *U.S. Strategic Nuclear Forces – Background, Developments, and Issues* (Congressional Research Service, February 2025).

41. Charles Perrow, *Normal Accidents: Living with High-Risk Technologies* (Princeton: Princeton University Press, 1984).

42. U.S. Senate Committee on Environment and Public Works, “Carper, Capito, Whitehouse Applaud Senate Passage of Nuclear Energy Bill, the ADVANCE Act,” 18 June 2024, <https://www.epw.senate.gov/public/index.cfm/2024/6/carper-capito-whitehouse-applaud-senate-passage-of-nuclear-energy-bill-the-advance-act> (1 April 2025).

43. Allison Macfarlane, “Trump Just Assaulted the Independence of the Nuclear Regulator. What Could Go Wrong?,” *Bulletin of the Atomic Scientists*, 21 February 2025, <https://thebulletin.org/2025/02/trump-just-assaulted-the-independence-of-the-nuclear-regulator-what-could-go-wrong/> (1 April 2025).

44. Donald Trump, “Establishing the National Energy Dominance Council,” *The White House*, February 2025, <https://www.whitehouse.gov/presidential-actions/2025/02/establishing-the-national-energy-dominance-council/> (1 April 2025).

45. Sonja D. Schmid, *The Pre-Chernobyl History of the Soviet Nuclear Industry* (Cambridge, MA: MIT Press, 2015), p.85.

46. Edwin Lyman, “Five Things the ‘Nuclear Bros’ Don’t Want You to Know About Small Modular Reactors,” *Union of Concerned Scientists*, 30 April 2024, <https://blog.ucsusa.org/edwin-lyman/five-things-the-nuclear-bros-dont-want-you-to-know-about-small-modular-reactors/> (1 April 2025).

47. Perrow, *Normal Accidents*, p.8 (note 41).

48. J. Samuel Walker, “Regulating Against Nuclear Terrorism: The Domestic Safeguards Issue, 1970–1979,” *Technology and Culture* 42 (2001): 107–32.

49. Rich McCormick, “Odds Are We’re Living in a Simulation, Says Musk,” *The Verge*, 2 June 2016, <https://www.theverge.com/2016/6/2/11837874/elon-musk-says-odds-living-in-simulation> (10 March 2025).

50. Brigid Kenney, “The Billionaire-Led Quest for Immortality,” *The Week*, 20 September 2023, <https://theweek.com/science/the-billionaire-led-quest-for-immortality> (10 March 2025).

51. Ryan Holiday, *Conspiracy: Peter Thiel, Hulk Hogan, Gawker, and the Anatomy of Intrigue* (New York: Penguin, 2018), p.30.

52. Laura Sydell, “Major Trump Backer’s Alleged Positive Comments about Apartheid Stir Anger,” *NPR All Tech Considered*, 3 November 2016, <https://www.npr.org/sections/alltechconsidered/2016/11/03/500569299/major-trump-backers-positive-comments-about-apartheid-stir-anger> (10 March 2025).

53. Max Chafkin, *The Contrarian: Peter Thiel and Silicon Valley’s Pursuit of Power* (New York: Penguin, 2021).

54. Fred Turner, *From Counterculture to Cyberculture: Stewart Brand, the Whole Earth Network, and the Rise of Digital Utopianism* (Chicago: University of Chicago Press, 2006).

55. Patrick McCray, *The Visioneers: How a Group of Elite Scientists Pursued Space Colonies, Nanotechnologies, and a Limitless Future* (Princeton: Princeton University Press, 2013); David Kaiser and W. Patrick McCray (eds.), *Groovy Science: Knowledge, Innovation, and American Counterculture* (Chicago: University of Chicago Press, 2016).

56. Joel N. Shurkin, *Broken Genius: The Rise and Fall of William Shockley, Creator of the Electronic Age* (Macmillan, 2006); Glenna Matthews, *Silicon Valley, Women, and the California Dream: Gender, Class, and Opportunity in the Twentieth Century* (Stanford: Stanford University Press, 2003); Noam Cohen, *The Know-It-Alls: The Rise of Silicon Valley as a Political Powerhouse and Social Wrecking Ball* (London: Simon & Schuster, 2018).

57. Christophe Lécuyer, *Making Silicon Valley: Innovation and the Growth of High Tech, 1930–1970* (Cambridge, MA: MIT Press, 2005), chapter 3; also Margaret O’Mara, *Cities of Knowledge: Cold War Science and the Search for the Next Silicon Valley* (Princeton: Princeton University Press, 2005), chapter 3.

58. Stuart W. Leslie, *The Cold War and American Science: The Military-Industrial-Academic Complex at MIT and Stanford* (New York: Columbia University Press, 1993), chapter 9.

59. Adam Rome, “‘Give Earth a Chance’: The Environmental Movement and the Sixties,” *Journal of American History* 90 (2003): 525–54; Naomi Oreskes and Erik Conway, *Merchants of Doubt: How a Handful of Scientists Obscured the Truth on Issues from Tobacco Smoke to Global Warming* (London: Bloomsbury Press, 2010), chapter 1.

60. Leslie Berlin, *The Man Behind the Microchip: Robert Noyce and the Invention of Silicon Valley* (Oxford: Oxford University Press, 2005).

61. The quote, and much of the rest of this article, are taken from Cyrus C. M. Mody, *The Squares: US Physical and Engineering Scientists in the Long Cold War* (Cambridge, MA: MIT Press, 2022), chapter 6.

62. Arnold Thackray, David C. Brock, and Rachel Jones, *Moore’s Law: The Life of Gordon Moore, Silicon Valley’s Quiet Revolutionary* (New York: Basic Books, 2015).

63. Christophe Lécuyer, “From Clean Rooms to Dirty Water: Labor, Semiconductor Firms, and the Struggle over Pollution and Workplace Hazards in Silicon Valley,” *Information and Culture* 52 (2017): 304–33.

64. Margaret O’Mara, *The Code: Silicon Valley and the Remaking of America* (New York: Penguin Books, 2019); Mariana Mazzucato, *The Entrepreneurial State: Debunking Public vs Private Sector Myths* (New York: Anthem Press, 2013).

65. EO 14168, “Defending Women from Gender Ideology Extremism and Restoring Biological Truth to the Federal Government,”emphasis added, 20 January 2025, <https://www.whitehouse.gov/presidential-actions/2025/01/defending-women-from-gender-ideology-extremism-and-restoring-biological-truth-to-the-federal-government/> (1 April 2025).

66. AP, “Transcript of President Donald Trump’s Speech to a Joint Session of Congress,” emphasis added, <https://apnews.com/article/trump-speech-congress-transcript-751b5891a3265ff1e5c1409c391fef7c>

67. David Wallace-Wells, “How Covid Remade America,” *The New York Times*, 4 March 2025.

68. Alice Domurat Dreger, *Hermaphrodites and the Medical Invention of Sex* (Cambridge, MA: Harvard University Press, 1998).

69. Nelly Oushoorn, *Beyond the Natural Body: An Archaeology of Sex Hormones* (New York: Routledge, 1984).

70. Sandra Eder, *How the Clinic Made Gender: The Medical History of a Transformative Idea* (Chicago: University of Chicago Press, 2022).

71. Alice Miller, “Your True and Proper Gender: The Barr Body as a Good Enough Science of Sex,” *Studies in the History and Philosophy of Biological and Biomedical Sciences* 37 (2006): 459–83.

72. Joe Hin Tjio and Albert Levan, “The Chromosomes Number in Man,” *Hereditas* 42 (1956): 1–6.

73. Peter Harper, *First Years of Human Chromosomes* (Oxfordshire: Scion, 2006).

74. Scott Atran, *Fondements de l’histoire naturelle* (Brussels: Editions Complexe, 1986).

75. Nicolas Copernicus, *On the Revolutions of the Heavenly Spheres* (Nuremberg, 1543).

76. AP, Transcript of President Donald Trump’s Speech (note 66). emphasis added.

77. On American Cold War science, see Audra Wolfe, *Competing with the Soviets: Science, Technology, and the State in Cold War America* (Baltimore, MD: Johns Hopkins University Press, 2013); Jessica Wang, *American Science in an Age of Anxiety: Scientists, Anticommunism, and the Cold War* (Chapel Hill: University of North Carolina Press, 1999).

78. Jessica M. Smith, Kacey Beddoes, Gary Downey, et al., “Editorial: We Have Been Here Before: Reflections on Engineering and Authoritarianism,” *Engineering Studies* 17, no. 1 (2025): 1–10, <https://www.tandfonline.com/doi/full/10.1080/19378629.2025.2466994#d1e375> (8 April 2025).

79. See Tiago Saraiva, *Fascist Pigs: Technoscientific Organisms and the History of Fascism* (Cambridge, MA: MIT Press, 2016).

80. Naomi Oreskes and John Krige (eds.), *Science and Technology in the Global Cold War* (Cambridge, MA: MIT Press, 2014); Gabrielle Hecht (ed.), *Entangled Geographies: Empire and Technopolitics in the Global Cold War* (Cambridge, MA: MIT Press, 2011).

81. Lee Zeldin, “EPA Ends the ‘Green New Deal’,” *The Wall Street Journal*, 12 March 2025, <https://www.wsj.com/opinion/lee-zeldin-epa-ends-the-green-new-deal-aa81de06?mod=opinion_lead_pos5> (8 April 2025).

82. Ibid.

83. Ariel Wittenberg, Chelsea Harvey, and E&E News, “Climate Researchers Wait for the Ax to Fall,” *Scientific American*, 28 March 2025, <https://www.scientificamerican.com/article/amid-trump-cuts-climate-researchers-wait-for-the-ax-to-fall/> (8 April 2025).

84. Joshua P. Howe, *Behind the Curve: Science and the Politics of Global Warming* (Seattle: University of Washington Press, 2016); Paul N. Edwards, *A Vast Machine: Computer Models, Climate Data, and the Politics of Global Warming* (Cambridge, MA: MIT Press, 2010); Jacob Darwin Hamblin, *Arming Mother Nature: The Birth of Catastrophic Environmentalism* (Oxford: Oxford University Press, 2013).

85. The quote is by Charles F. Kennel of the Scripps Institution of Oceanography. American Chemical Society, “National Historic Chemical Landmarks: The Keeling Curve,” 2015, <https://www.acs.org/content/dam/acsorg/education/whatischemistry/landmarks/keeling-curve/2015-keeling-curve-landmark-booklet.pdf> (8 April 2025).

86. Gloria Dickie, “Trump Cuts Target Mauna Loa Observatory,” *Hawaii Tribune Herald*, 4 March 2025, <https://www.hawaiitribune-herald.com/2025/03/11/hawaii-news/trump-cuts-target-world-leading-greenhouse-gas-observatory-in-hawaii/> (14 April 2025).

87. On climate skepticism, see Naomi Oreskes and Erik M. Conway, *Merchants of Doubt: How a Handful of Scientists Obscured the Truth Issues from Tobacco Smoke to Global Warming* (London: Bloomsbury, 2010); Mike Hulme, *Why We Disagree About Climate Change: Understanding Controversy, Inaction and Opportunity* (Cambridge: Cambridge University Press, 2009).

88. John R. McNeill and Corinna R. Unger (eds.), *Environmental Histories of the Cold War* (Cambridge: Cambridge University Press, 2010).

89. Carola Sachse and Mark Walker (eds.), *Politics and Science in Wartime: Comparative International Perspectives on the Kaiser Wilhelm Institutes* [*Osiris*, 20] (Chicago: University of Chicago Press, 2005); Susanne Heim, Carola Sachse, and Mark Walker (eds.), *The Kaiser Wilhelm Society Under National Socialism* (Cambridge: Cambridge University Press, 2009); Mark Walker, *Hitler’s Atomic Bomb: History, Legend, and the Twin Legacies of Auschwitz and Hiroshima* (Cambridge: Cambridge University Press, 2024).

90. Mark Walker (ed.), *Science and Ideology: A Comparative History* (New York: Routledge, 2003).

91. See for example Mitchell G. Ash, “Wissenschaft und Politik. Eine Beziehungsgeschichte im 20. Jahrhundert,” *Archiv für Sozialgeschichte* 50 (2010): 11–46.

92. For the astonishing claim that there is still a debate on whether there is a constitutional crisis, see Amber Phillips, “The 5-Minute Fix: Why Experts Say Trump has Caused a Constitutional Crisis,” *The Washington Post Online*, 13 February 2025, <https://www.washingtonpost.com/video/politics/why-experts-say-trump-has-caused-a-constitutional-crisis/2025/02/13/887d9d75-47cb-4d55-812f-b88ecc57b537_video.html> (31 March 2025).

93. Trump v. United States, 603 U.S. 593 (2024).

94. As organized by 50501 (<https://www.fiftyfifty.one/>) and Indivisible (<https://indivisible.org>).

95. David Remnick, “We Are Sleepwalking Into Autocracy” (Interview with Senator Chris Murphy of Connecticut), *The New Yorker*, 30 March 2025, <https://www.newyorker.com/podcast/the-new-yorker-radio-hour/senator-chris-murphy-this-is-how-democracy-dies-everybody-just-gets-scared> (31 March 2025).

96. See, for example, Jeffrey Herf, “We Are Uncomfortably Close to 1933,” *Persuasion*, 20 March 2025. <https://www.persuasion.community/p/we-are-uncomfortably-close-to-1933> (31 March 2025); “Adam Gopnik on Hitler’s Rise to Power” (Podcast with David Remnick), *The New Yorker Online*, 25 March 2025, <https://www.newyorker.com/podcast/political-scene/adam-gopnik-on-hitlers-rise-to-power> (31 March 2025). For comparisons with tactics employed by authoritarians to seize power elsewhere, see also the postings by Timothy Snyder in his Substack blog “Thinking About,” <https://snyder.substack.com/> (31 March 2025).

97. The White House, “Restoring Truth and Sanity to American History,” 2025, <https://www.whitehouse.gov/presidential-actions/2025/03/restoring-truth-and-sanity-to-american-history/> (27 March 2025).

98. Steven Lee Myers and Stuart A. Thompson, “Falsehoods Drive Policy in Trump’s Second Term,” *The New York Times International Edition*, 28 March 2025, p.1, 8.

99. EPA Press Office, “EPA Launches Biggest Deregulatory Action in U.S. History,” 12 March 2025, <https://www.epa.gov/newsreleases/epa-launches-biggest-deregulatory-action-us-history> (2 April 2025).

100. Nation, “As NOAA Braces for More Cuts, Scientists Say Public Safety is at Risk,” 14 March 2025, <https://www.pbs.org/newshour/nation/as-noaa-braces-for-more-cuts-scientists-say-public-safety-is-at-risk> (2 April 2025).

101. Mitchell G. Ash, “Was heißt ‘Politisierung der Universität’?,” in Martin Kitzinger, Wolfgang Eric Wagner, and Ingo Runde (eds.), *Hochschule und Politik–Politisierung der Universität vom Mittelalter bis zur Gegenwart* (Basel: Schwabe 2022).

102. Josh Gruenbaum, Sean R. Keveny, and Thomas R. Wheeler (Anti-Semitism Task Force, Department of Education) to Alan M. Garber (President of Harvard University) and Penny Pritzker (Lead Member, Harvard Corporation), 11 April 2025, published in *The New York Times*, 14 April 2025, <https://www.nytimes.com/interactive/2025/04/14/us/trump-harvard-demands.html> (20 April 2025); Alan M. Garber to Members of the Harvard Community, 14 April 2025. Received via personal communication, 14 April 2025.

103. Alan Blinder, Anemona Hartocollis, Vimal Patel, and Stephanie Saul, “Why Harvard Decided to Fight Trump.” *The New York Times*, 15 April 2025, <https://www.nytimes.com/2025/04/15/us/why-harvard-resisted-trumps-demands.html> (20 April 2025).

104. See, for example, Distraught, “Stand Up for Science this Friday. Over 100 Protests Nationwide,” *Daily Kos*, 5 March 2025, <https://www.dailykos.com/stories/2025/3/5/2308034/-Stand-Up-For-Science-this-Friday-over-100-protests-nationwide> (31 March 2025).

105. Michael S. Roth, “Making Free Speech Meaningful,” *Wesleyan Magazine*, 7 April 2025.

106. “Historians Defend the Smithsonian,” 31 March 2025, cosigned by 32 organizations thus far <https://www.historians.org/news/historians-defend-the-smithsonian/>; “AHA–OAH Joint Statement on Federal Censorship of American History,” 13 March 2025, cosigned by 40 organizations, <https://www.historians.org/news/aha-oah-joint-statement-on-federal-censorship-of-american-history/>; “American Historical Association Condemns Indiscriminate Cuts to the Federal Government,” 24 March 2025, cosigned by 19 organizations, <https://www.historians.org/news/american-historical-association-condemns-indiscriminate-cuts-to-the-federal-government/> (31 March 2025). The History of Science Society has yet to issue any statement, but has published extensive information from AAAS on its web site: <https://hssonline.org/news/694847/Information-for-HPSTM/Science-Studies-Community-Regarding-Current-Attacks-on-Science-26-Feb-2025.htm> (31 March 2025).

107. H. Holden Thorp, “Come Together, Right Now,” *Science* 387 (2025): 909; Sai Teja Boppinisti, “AAAS Leadership Speaks Out Against Trump Administration’s Attacks on Science,” 18 February 2025, <https://associationofresearch.org/aaas-leadership-speaks-out-against-trump-administrations-attacks-on-science/> (20 April 2025).

108. Molly Galvin, interview with Marcia McNutt, “America Can’t Be Great Without Great Science. That is Where the Academies Can Help,” 10 March 2025, <https://www.nationalacademies.org/news/2025/03/america-cant-be-great-without-great-science-that-is-where-the-academies-can-help> (31 March 2025).

109. Jessica Glenza, “More Than 1,900 Scientists Write Letter in ‘SOS’ over Trump’s Attacks on Science,” *The Guardian*, 31 March 2025, <https://www.theguardian.com/us-news/2025/mar/31/scientists-letter-trump-administration> (1 April 2025).

110. Maya C. Miller, “Foregoing Oversight, Republicans Call Musk,” *The New York Times International Edition*, 11 March 2025, p.6.

111. Gabrielle Masson, “European Countries, Universities Implement Initiatives to Attract US Researchers Amid ‘Brutal Funding Cuts’,” *Fierce Biotech*, 21 March 2025.

112. Aric Sleeper, “Hundreds gather at UC Santa Cruz to Stand Up for Science,” *Santa Cruz Sentinel*, 7 March 2025, <https://www.santacruzsentinel.com/2025/03/07/hundreds-gather-at-uc-santa-cruz-to-stand-up-for-science/> (30 March 2025).

113. In February, 2025, a diverse group of Duke University faculty, staff, and students organized against depredations by the U.S. federal administration against people and programs. Many action ideas fill their regularly updated document, “Duke People Action Items.” A principal demand is that Duke not comply with federal orders to dismantle diversity, equity, and inclusion efforts. They note that Duke has an endowment that allows the institution to take risks. Their email user name is “careassembly.” In the face of so much anticipatory obedience throughout U.S. institutions, I find these university colleagues/companions to be models for refusing obedience to unjust power. See Rebecca Fan and Sarah Diaz, “‘Duke, Don’t Comply’: Duke, Durham Community Members Protest to Demand University Stand Up to Trump, End PPD Policy,” *The Chronicle*, 26 February 2025, <https://www.dukechronicle.com/article/2025/02/duke-chronicle-duke-dont-comply-protest-trump-administration-dei-ppd-policy-east-campus-demonstration-diversity-equity-inclusion-free-speech-activism> (30 March 2025).

114. Martin Heidegger, “Rectoratsrede,” “The Self-Assertion of the German University: Address, Delivered on the Solemn Assumption of the Rectorate of the University Freiburg,” *The Rectorate 1933/34: Facts and Thoughts*. Translated with an Introduction by Karsten Harries, *Review of Metaphysics* 38, no. 3 (1985): 1–36. <https://archive.org/details/MartinHeidegger-TheSelfAssertionOfTheGermanUniversity1933> (30 March 2025).

115. For the text of the letter sent to Columbia’s interim president and co-chairs of the board of trustees from the Trump regime on 13 March 2025, see “Read the Letter to Columbia University,” *The New York Times*, 14 March 2025, <https://www.nytimes.com/interactive/2025/03/14/nyregion/columbia-letter.html> (30 March 2025). For the text of Columbia’s capitulation by the interim president, see “Fulfilling Our Commitments,” April 2025, <https://president.columbia.edu/content/fulfilling-our-commitments> (30 March 2025). This response did not save her position. She resigned on 28 March 2025, succumbing to ongoing pressure and mistrust from the Trump regime.

116. The controversial definition of antisemitism now used by Columbia in practice <https://holocaustremembrance.com/resources/working-definition-antisemitism> (30 March 2025) discourages or bans most organized calls for boycotts, divestment, and sanctions, and much opposition to Israeli war policies, occupations, and crimes against humanity in Gaza and the West Bank. Task Force on Antisemitism, Report #2, *Columbia University Student Experiences of Antisemitism and Recommendations for Promoting Shared Values and Inclusion*, 24 August 2024, p.45. This report vividly describes many experiences of antisemitism at Columbia that unquestionably require strong action. However, another equally controversial definition of antisemitism exists that could be fruitfully used if actually rooting out and punishing violence and harassment against Jews were the objective. “Jerusalem Declaration on Antisemitism,” March 2021, <https://jerusalemdeclaration.org/> (30 March 2025).

117. For over a dozen constitutional scholars’ analysis of the illegal misuse by the Trump administration of Article VI of the Civil Rights Act in its claims of antisemitism without the full investigation and due process required by Article VI, see Eugene Volokh, Michael C. Dorf, David Cole, et al., “A Statement from Constitutional Law Scholars on Columbia,” *The New York Review of Books*, 20 March 2025, <https://www.nybooks.com/online/2025/03/20/a-statement-from-constitutional-law-scholars-on-columbia/?utm_source=nybooks&utm_medium=email&utm_campaign=email-share> (30 March 2025).

118. Excerpts are from a draft letter to *The Chronicle of Higher Education* signed by faculty around the country in March 2025. The letter responds to more than 60 colleges and universities receiving warning letters from the Department of Education threatening extortion similar to withholding $400 million in funding by the federal government if Columbia did not comply. Columbia and its futile compliance (further threats ensued) were to be object lessons of what was coming for others. Ronak D. Desai and Daniel Prince, “Trump Administration Investigates 60 Universities for Antisemitism Amid Escalating Federal Oversight of Higher Education: Key Takeaways for Higher Ed Leaders,” *Paul Hastings*, 12 March 2025, <https://www.paulhastings.com/insights/client-alerts/trump-administration-investigates-60-universities-for-antisemitism-amid-escalating-federal-oversight-of-higher-education> (30 March 2025).

119. Affirming fundamental Catholic and Jesuit values and refusing to obey improper and likely unconstitutional orders to eliminate diversity, equity, and inclusion, the 6 March 2005 letter by William M. Treanor, Dean and Executive Vice President of Georgetown Law, is soberly inspiring. Many such letters are being written. I want more collective public outcry, but it is taking shape.

120. There are a few honorable exceptions to the anticipatory obedience and capitulations. Michael Roth of Wesleyan University tried and failed to bring together university presidents for a joint strong statement. Only the president of Princeton joined Roth in criticizing the assault on academic freedom and Columbia’s response to it. Christopher Eisgruber, “The Cost of the Government’s Attack on Columbia,” *The Atlantic*, 19 March 2025, <https://www.theatlantic.com/ideas/archive/2025/03/columbia-academic-freedom/682088/> (30 March 2025). Similar to my comparison of the Nazi moment for the German university in 1933 to Columbia University in 2025, Roth called the present conjuncture in the United States a “Vichy moment,” after the establishment of the collaborationist Vichy regime in 1940. See Robert Kuttner, “Columbia’s Capitulation and Wesleyan’s Pushback,” *American Prospect*, 24 March 2025, <https://prospect.org/blogs-and-newsletters/tap/2025-03-24-columbias-capitulation-wesleyans-pushback/> (30 March 2025). Also, Rich Lyons, Chancellor, and Ben Hermalin, Executive Vice Chancellor and Provost of the University of California Berkeley (UCB), issued a strong statement in a mass email to UCB people regarding a rally held on campus on 17 March 2025. Facebook post by Paola Bacchetta, 17 March 2025 (28 March 2025). Beginning April 14, 2025, Harvard University fought back hard, refusing to comply with intrusive federal orders or lose billions of dollars in grants. Hopefully, many more will follow, <https://news.harvard.edu/gazette/story/2025/04/harvard-wont-comply-with-demands-from-trump-administration/> (16 April 2025).

121. Steven Shapin and Simon Schaffer, *Leviathan and the Air-Pump* (Princeton: Princeton University Press, 1985).

122. Donna Haraway, “The Transformation of the Left in Science: Radical Associations in Britain in the 30’s and the U.S.A. in the 60’s,” *Soundings* 58, no. 4 (1975): 441–62. Gary Werskey, *The Visible College: A Collective Biography of British Scientists and Socialists of the 1930s* (New York: Holt, Rinehart and Winston, 1979).

123. “Founding Document: 1968 MIT Faculty Statement,” December 1968, <https://www.ucs.org/about/history/founding-document-1968-mit-faculty-statement> (30 March 2025). The Founding Document was signed by 50 senior MIT faculty members, including the chairs of the biology, chemistry, and physics departments.

124. Sandra Kazahn Masur, “Women in Cell Biology: A Seat at the Table and a Place at the Podium,” *Molecular Biology of the Cell* 24, no. 2 (2013): 57–60.

125. Sandra Harding, *The Science Question in Feminism* (Ithaca, NY: Cornell University Press, 1986).

126. Donna Haraway, “Situated Knowledges: The Science Question in Feminism as a Site of Discourse on the Privilege of Partial Perspective,” *Feminist Studies* 14, no. 3 (1988): 575–99.

127. Sandra Harding, “Latin American Decolonial Social Studies of Scientific Knowledge: Alliances and Tensions,” *Science, Technology, & Human Values* 41, no. 6 (2016): 1063–87.

